# Global regulation and virulence mediated by the histidine-responsive local transcription factor HutC in *Pseudomonas aeruginosa*

**DOI:** 10.1128/mbio.03886-25

**Published:** 2026-02-05

**Authors:** Kiran S. Jayan, Naran Naren, Yunhao Liu, Xinyi Liu, Chang Luan, Xue-Xian Zhang

**Affiliations:** 1 School of Natural Sciences, Massey University 168219, Auckland, New Zealand; 2 Northeast Institute of Geography and Agroecology, Chinese Academy of Sciences 66276, Changchun, China; 3 School of Biological Sciences, University of Auckland, Auckland, New Zealand; Center for Microbial Dynamics & Infection, Atlanta, Georgia, USA

**Keywords:** *Pseudomonas aeruginosa*, histidine, urocanate, transcriptional factor, EMSA, DNase I footprinting, HutC, bacterial pathogenesis

## Abstract

Host-derived nutritional substrates fuel infection in invading bacteria, yet their potential as signaling molecules for host perception remains largely unexplored. Here, we report the functional characterization of HutC, a transcriptional repressor of *hut* genes for the utilization of histidine and its derivative, urocanate, in the human pathogenic bacterium *Pseudomonas aeruginosa* PAO1. Using electrophoretic mobility shift assay (EMSA) and DNase I footprinting combined with site-directed mutagenesis, we demonstrate that HutC specifically binds with high affinity to the promoters of the two *hut* operons. This analysis led to the identification of a noncanonical HutC-binding site in the *hutF* promoter of a non-pathogenic *Pseudomonas* strain, which is absent in *P. aeruginosa*. A genome-wide search of the PAO1 genome using a probability matrix of the canonical HutC-binding motif identified 172 candidate sites, many associated with bacterial pathogenesis. Their predicted low-affinity binding was experimentally validated by EMSA for six selected targets, including the aminoglycoside response regulator (*arr*). Deletion of *hutC* resulted in increased tobramycin-induced biofilm formation and impaired production of pyoverdine, an iron-scavenging siderophore. Moreover, the *hutC* mutant exhibited altered motility and significantly reduced virulence in the *Caenorhabditis elegans* infection model. Finally, transcriptome sequencing of three genetically distinct *hutC* mutants provided further support for the HutC-mediated global regulation. Together, these findings highlight the functional significance of low-affinity DNA binding by this transcription factor and support the hypothesis that HutC mediates *P. aeruginosa* virulence, with histidine and urocanate as effectors. Thus, HutC may represent a potential therapeutic target within the bacterial host-perception system.

**IMPORTANCE:**

*Pseudomonas aeruginosa* is a metabolically versatile environmental pathogen whose virulence relies on coordinated expression of catabolic genes, particularly the histidine utilization (*hut*) operon. Disruption of the *hut* operon reduces virulence, but the underlying mechanism remains rudimentary. Here, we genetically characterized the histidine-responsive transcriptional factor HutC in *P. aeruginosa* PAO1, alongside HutC in the non-pathogenic strain *Pseudomonas fluorescens* SBW25. Two important features emerged. First, HutC recognizes two distinct DNA-binding motifs with little sequence similarity; notably, a noncanonical-binding site was identified in the *hutF* promoter of SBW25 but was absent in PAO1. Second, HutC exhibits low-affinity binding to genes beyond histidine catabolism and contributes to the expression of multiple virulence traits. These findings identify HutC as a local regulator linking histidine catabolism with virulence and as a unique prokaryotic model for studying how noncanonical transcriptional factor-DNA interactions achieve binding specificity, a phenomenon so far investigated only in eukaryotes.

## INTRODUCTION

Bacterial infection of eukaryotic hosts is driven by the availability of nutrients provided by host cells, but this process is countered by host immune responses. While the importance of nutrients in bacterial pathogenesis has long been recognized, research has primarily focused on tissue-specific metabolic adaptation (1–3). The host is often regarded as a growth medium that supports bacterial survival and proliferation during infection (4, 5). However, nutritional substrates may play far more critical roles than previously assumed. In addition to supporting growth, host-derived metabolites can function as components of host-associated molecular patterns (HAMPs), enabling pathogenic bacteria to express virulence factors at the appropriate time and place to evade host immunity (6–9). In this regard, the absence or limited availability of particular metabolites may also serve as a host-specific signal that triggers the switching to a virulent state (10). Therefore, the mechanism of HAMP recognition could offer a new avenue for antibacterial intervention in the prevention of infectious disease (7, 11).

**Fig 1:**
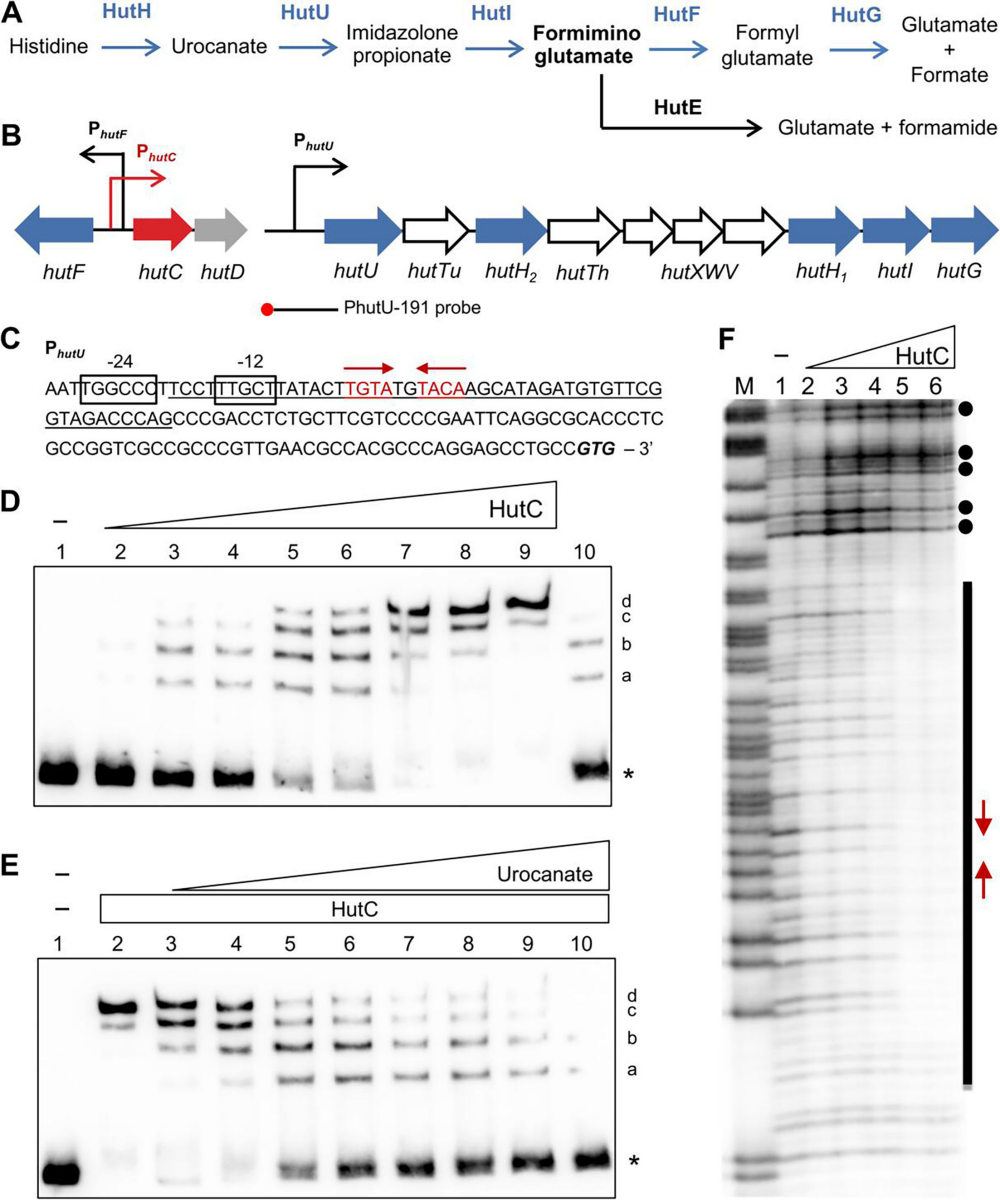
Histidine catabolism in *P. aeruginosa* and the specific interactions between HutC and the P*_hutU_* promoter DNA. (**A**) Histidine degradation pathways with a branch for the breakdown of formiminoglutamate (FIGLU). HutE is absent in *Pseudomonas fluorescens* SBW25. (**B**) Organization of *hut* genes in three transcriptional units. The biotin-labeled 5’-end of the probe is marked with a red circle. (**C**) DNA sequence of P*_hutU_* promoter upstream of the *hutU* start codon GTG. The HutC-protected region is underlined, and the conserved Phut-I and Phut-II elements are indicated by inverted arrows. (**D**) EMSA of His_6_-tagged HutC with PhutU-191 probe DNA. HutC was added at increasing concentrations of 0, 70, 140, 200, 300, 400, 600, 800, and 1200 nM in lanes 1 to 9, respectively. In lane 10, 800 nM HutC was mixed with the unlabeled probe at a 75-fold molar excess. The position of the free probes is indicated by an asterisk, and the four shifted bands are denoted by a, b, c, and d. (**E**) EMSA of HutC (800 nM) with urocanate added at increasing concentrations of 0, 0.05, 0.1, 0.2, 0.3, 0.4, 0.6, 1.0, and 1.5 mM in lanes 2 to 10, respectively. Lane 1 is a free probe-only control. (**F**) DNase I footprinting conducted with His_6_-tagged HutC and biotin-labeled PhutU-191 probe. Lane M, G+A marker; lanes 1-6, HutC added at increasing concentrations from 0, 0.5, 1.0, 2.0, 4.0, to 6.0 µM. The HutC-protected region, Phut sites, and hypersensitive DNase I cleavage sites are shown in black bar, inverted arrows, and filled circles, respectively.

Mounting evidence suggests that histidine, or more likely its derivative urocanate, is such a potential HAMP molecule crucial for bacterial infection (6). As illustrated in Fig. 1A, urocanate is produced from histidine by the enzyme histidase (HutH, EC 4.3.1.3) and subsequently metabolized by urocanase (HutU, EC 4.2.1.49) (12). These two enzymatic steps are highly conserved from bacteria to plants, animals, and humans, implying that urocanate could accumulate in tissues lacking urocanase activity (13). Urocanate is known to act as a natural sunscreen in human skin, where it accumulates and protects against ultraviolet radiation (13). Intriguingly, urocanate serves as a signaling compound for eyeless, skin-penetrating parasitic nematodes to locate their hosts (14). Thus, a similar host-perception mechanism may have evolved in environmental pathogenic bacteria, such as *Pseudomonas aeruginosa*, a causative agent of opportunistic infections in immunocompromised individuals, including pneumonia, urinary tract infections, and wound infections (15, 16). Indeed, the histidine catabolism is linked to the virulence of *P. aeruginosa* PAO1, although the underlying mechanism remains largely unknown (17).

Our previous work on the regulation of histidine utilization (*hut*) genes focused on *Pseudomonas fluorescens* SBW25, a non-pathogenic, plant-associated model strain (18). The SBW25 genome encodes a similar set of structural genes for histidine uptake and degradation, organized in a manner similar to that of *P. aeruginosa*, except that *P. aeruginosa* strains possess an alternative pathway for the enzymatic breakdown of formiminoglutamate (FIGLU; Fig. S2A and B) (19). Expression of the *hut* operons is induced by histidine under the control of the HutC repressor (Fig. 1B), with urocanate—rather than histidine—acting as the effector molecule (20). HutC is a representative member of the GntR/HutC family of transcriptional regulators, characterized by an N-terminal winged helix-turn-helix DNA-binding domain and a C-terminal substrate-binding domain. Its mode of action was examined in detail at the *hutU* promoter (P*_hutU_*) of SBW25, using electrophoretic mobility shift assay (EMSA) and DNase I footprinting (21). HutC recognizes and binds to an operator site centered on a consensus sequence of TGTA-N_2_-TACA (Phut). Moreover, HutC specifically targets a distinct Pntr site in the *ntrBC* promoter, and this low-affinity binding represents a feedback loop essentially required for NtrBC to directly activate expression of the *hutU* operon when growing on histidine as a nitrogen source (22). Consequently, as a key mediator for sensing histidine catabolic rate, *hutC* is tightly autoregulated and is organized together with *hutF* via overlapping promoters in *Pseudomonas* strains that harbor the five-step *hut* pathway (Fig. 1B; Fig. S2B). In *P. fluorescens* SBW25, HutC is also functionally required for competitive colonization *in planta*, despite the fact that histidine is not a major nutrient on plant surfaces (20, 21, 23).

Here, we extend our investigation to *P. aeruginosa*, an important human pathogen that also causes disease in various plants and animals (24). Our work aims to provide evidence supporting the hypothesis that HutC acts as a mediator of virulence in *P. aeruginosa* in response to histidine or urocanate in the host environment (6). Building on our previous work on HutC interactions with the P*_hutU_* promoter in SBW25, we first examined the specific binding of purified HutC protein with the P*_hutU_* promoter DNA in *P. aeruginosa* PAO1. This was followed by a comparative EMSA and DNase I analyses of HutC interactions with the P*_hutF_* promoter in both PAO1 and SBW25. Upon identifying a noncanonical HutC-binding site in P*_hutF_*, we performed a genome-wide search for HutC target sites in the genome of PAO1. A panel of six candidate sites was subsequently verified by EMSA or DNase I footprinting. Next, we investigated the phenotypic effects of *hutC* deletion on *P. aeruginosa* virulence and virulence-associated traits, including antibiotic-induced biofilm formation, motility, and iron-scavenging siderophore production. Finally, transcriptome sequencing was performed to uncover the global changes of gene expression caused by *hutC* genetic alteration (i.e., *hutC* deletion, *hutC* located at a separate *att*Tn7 site, and *hutC* constitutive overexpression [OE]). Our data indicate that the local transcription factor (TF) HutC exerts global regulatory effects beyond histidine catabolism and contributes to the regulation of *P. aeruginosa* virulence. The functional and molecular significance of the noncanonical HutC binding will also be discussed.

## RESULTS

### HutC displays high-affinity binding with the *hutU* promoter in *P. aeruginosa*

The *hutF* gene is positioned separately from the rest of the *hut* structural genes by sharing the same operator site employed by HutC for autoregulation (Fig. 1B). This arrangement suggests that *hutF* is more tightly controlled by HutC than *hut* genes within the *hutU-hutG* operon, a distinction that is likely crucial for preventing intracellular accumulation of the toxic intermediate FIGLU (Fig. 1A). To test this hypothesis, the DNA-binding properties of His_6_-tagged HutC from *P. aeruginosa* PAO1 (hereafter, HutC_PAO1_) were examined using EMSA and DNase I footprinting with *hutU* and *hutF* promoters. More specifically, the specific probe DNA containing 191 bp of the *hutU* promoter (P*_hutU_*) region was amplified by PCR using a biotin-labeled primer (Table S2). As shown in Fig. 1, four shifted bands were gradually observed along with the increase of HutC_PAO1_ concentrations (Fig. 1D, lanes 2–9). DNA retardation was mostly eliminated with the addition of excess unlabeled PhutU-191 probe as the competitor DNA (Fig. 1D, lane 10). Next, the effects of urocanate were investigated by maintaining a constant HutC_PAO1_ protein concentration while varying concentrations of urocanate (Fig. 1E). As expected, a decrease in shifted bands coupled with an increase in unbound probe DNA was observed upon the addition of increasing concentrations of urocanate (lanes 2–10). Together, the EMSA data revealed DNA-binding properties of HutC_PAO1_ similar to HutC in *P. fluorescens* SBW25 (i.e., HutC_SBW25_) (21) with their respective P*_hutU_* promoters. More specifically, HutC_PAO1_ also exhibits four oligomeric states and can sequentially form a monomer, dimer, trimer, and tetramer as its concentration increases, thereby achieving the maximum level of P*_hutU_* repression.

Finally, a DNase I footprinting assay was performed using purified HutC_PAO1_ and the PhutU-191 probe DNA (Fig. 1F). Results revealed that HutC_PAO1_ protects a 51 bp region in the *hutU* promoter of PAO1, centered around the previously identified canonical Phut site for HutC binding, with the consensus sequence of TGTA-N_2_-TACA (Fig. 1C). The HutC_PAO1_-protected region overlaps with the σ^54^ target site, which explains the repressor role of HutC in histidine-induced expression of the *hutU-G* operon.

### HutC displays moderate-affinity binding with the *hutF* promoter in *P. aeruginosa*

The *hutF* gene is divergently transcribed from the *hutCD* operon (Fig. 1B), and a putative HutC target site (Phut) is present in the intergenic region of *hutF* and *hutC*, suggesting that HutC binds to a shared operator to mediate histidine-induced expression of both *hutF* and *hutC*. However, the predicted HutC-binding activity has not yet been experimentally examined in *Pseudomonas*, including *P. fluorescens* SBW25 and *P. aeruginosa* PAO1. To this end, we first performed EMSA using His_6_-tagged HutC_PAO1_ and a 176 bp biotinylated probe of the PAO1 *hutF* promoter (PhutFC-176). Unlike P*_hutU_*, a significant shift of only one band was observed, even when HutC_PAO1_ was added at the highest concentration of 2.0 μM (Fig. S1A). Subsequent EMSA confirmed the role of urocanate in the dissociation of HutC–P*_hutF_*-specific binding in a concentration-dependent manner (Fig. S1B).

Next, a continuous variation analysis, or Job plot, was used to determine the stoichiometry of the specific HutC–P*_hutF_* interactions in PAO1. This time, EMSA was conducted with varying molar ratios of HutC_PAO1_ to the PhutFC-176 probe DNA, while their total concentrations were kept constant at 400 nM (Fig. S1C). Intensities of the shifted bands were plotted against HutC_PAO1_ fraction of the protein/DNA complex. The intersection point of the rising and falling subsets of the data produced a HutC_PAO1_ fraction of 0.650, corresponding to a DNA:protein ratio of 1:1.87 (Fig. S1D), and suggesting that the single shifted band detected in EMSA represents a dimer.

Together, the EMSA data described above indicate that HutC_PAO1_ binds to P*_hutF_* as a homodimer, whereas higher-order oligomerization occurs at the P*_hutU_* promoter region. The dissociation constant (*K_d_*) was calculated to be 250 ± 13.09 nM for P*_hutU_* and 1.34 ± 0.11 μM for P*_hutF_*. These findings suggest that, in the absence of the urocanate effector, HutC exerts stronger repression on P*_hutU_* than on P*_hutF_* in *P. aeruginosa*.

### Identification of a noncanonical HutC target site in the *hutF* promoter of *P. fluorescens* but absent in *P. aeruginosa*

The specific HutC–P*_hutF_* interactions were investigated in parallel by EMSA in a non-pathogenic model bacterium *P. fluorescens* SBW25, using His_6_-tagged HutC_SBW25_ and a 256 bp biotin-labeled probe (PhutFC-256), spanning −142 to +114 of the *hutF* gene (Fig. S2B). To our surprise, three predominant shifted bands were observed when HutC_SBW25_ was added at higher concentrations (Fig. S2C), and subsequent EMSA confirmed the predicted dissociation of the three protein/DNA complexes mediated by increasing concentrations of urocanate (Fig. S2D). Stoichiometric analysis using Hilmar Bading’s method (25) suggests that the three shifted bands represent a dimer, tetramer, and hexamer (Fig. S2E). Consequently, a weak band occasionally detected between bands “a” and “b” most likely represents a trimer (Fig. S2C).

**Fig 2:**
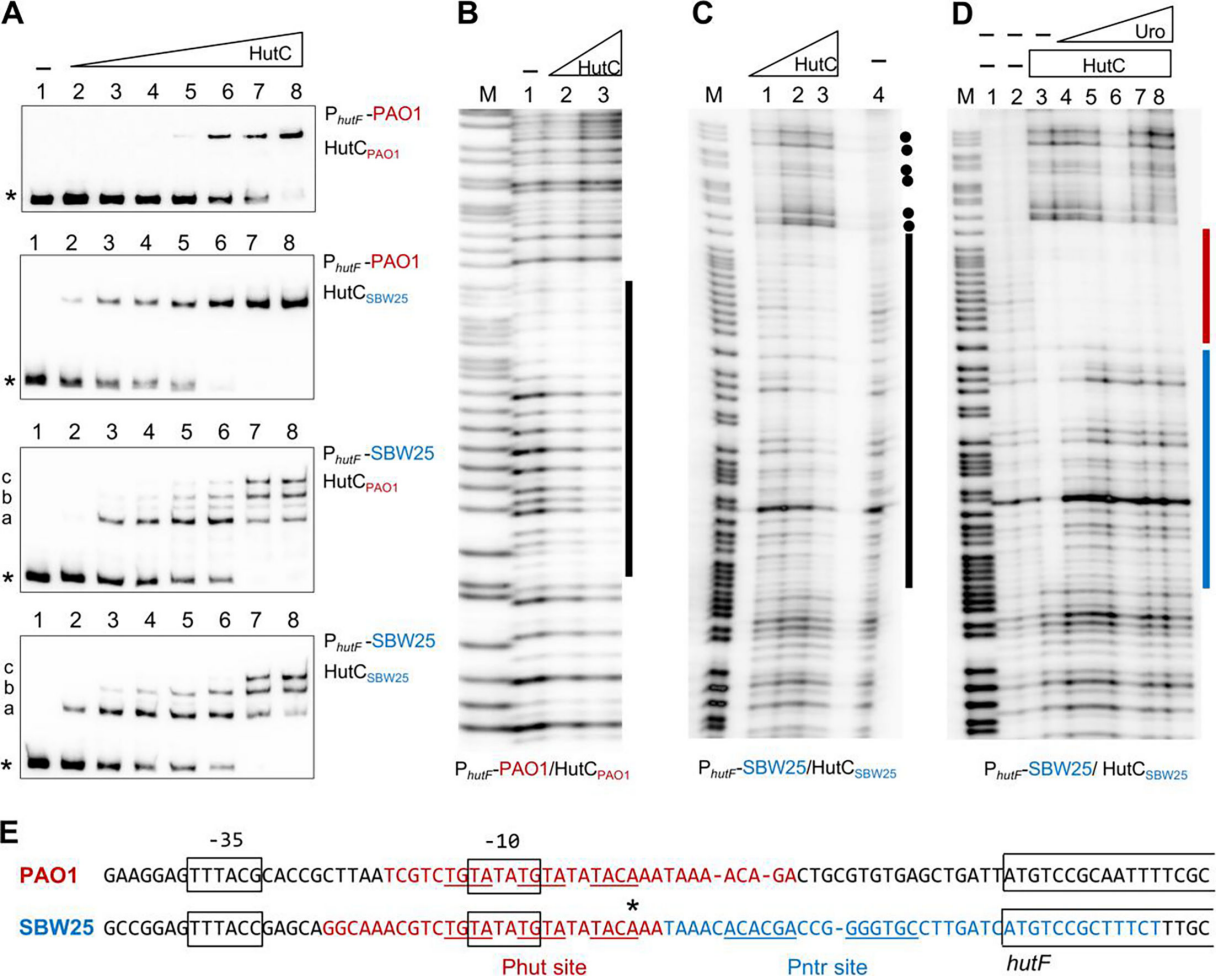
Identification of distinct HutC-binding sites in the P*_hutF_* promoters. (**A**) EMSAs performed using HutC proteins and P*_hutF_* promoter DNAs across *P. aeruginosa* PAO1 and *P. fluorescens* SBW25. The species origin of HutC protein and P*_hutF_* probe DNA is indicated to the right of each gel image. HutC was added at increasing concentrations of 0, 70, 140, 200, 300, 400, 600, and 800 nM in lanes 1–8, respectively. (**B**) DNase I footprinting of PAO1 HutC-P*_hutF_* interactions. HutC was added at 0, 500, and 2,000 nM in lanes 1–3. (**C**) DNase I footprinting of SBW25 HutC-P*_hutF_* interactions. HutC was added in lanes 1–3 at 1.4, 2.8, and 4.2 µM. Lane 4 is a no-protein control. (**D**) Effects of urocanate were examined in lanes 3–8 at increasing concentrations of 0, 0.5, 1, 2, 3, and 5 mM, containing HutC at the same concentration of 2.8 µM. Lanes 1 and 2 are negative control without protein and urocanate. M, G+A marker. HutC-protected regions are marked by bars on the right. Black dots denote hypersensitive DNase I cleavage sites. (**E**) Sequence alignment of P*_hutF_* promoters in strains PAO1 and SBW25. Strong and weak HutC-protected regions are indicated in red and blue font, respectively. The conserved Phut and Pntr elements are underlined. Asterisk denotes transcription start site previously determined in SBW25.

Given the high sequence similarity between HutC_PAO1_ and HutC_SBW25_ (84.3% identity) and the presence of a single palindromic target site (Phut) in both P*_hutF_* promoters (Fig. 2E), the distinct EMSA profiles described above raised important questions regarding the underlying molecular mechanisms—specifically, whether the differences were attributable to the HutC protein and/or the *hutF* promoter. To address this, we conducted gel shift assays using HutC protein and P*_hutF_* probe DNA across the two *Pseudomonas* species. As shown in Fig. 2A, only a single shifted band was observed for the P*_hutF_* probe from PAO1 (top two gel images), whereas three shifted bands appeared for the P*_hutF_* probe from SBW25 (bottom two gel images), regardless of the species origin of the purified HutC proteins. These results clearly indicate that the observed differences in HutC-P*_hutF_* interactions between PAO1 and SBW25 are attributable solely to the promoter DNA sequences.

Next, DNase I footprinting was conducted to identify the specific HutC-binding sequences in the *hutF* promoters of PAO1 and SBW25. A 32 bp region was protected from DNase I digestion in the PAO1 P*_hutF_* promoter (Fig. 2B), whereas a substantially larger 68 bp region was protected in the SBW25 P*_hutF_* promoter (Fig. 2C). Both protected regions overlap with their respective σ^70^ recognition sites; however, in SBW25, the HutC-binding sequence extends into the *hutF* coding region (Fig. 2E). Notably, this region contains an imperfect palindrome (ACACGAccgGGGTGC), which resembles the consensus sequence of NtrC-binding sites in *Pseudomonas* (GCACCA-N_3_-TGGTGC) (22, 26). This site is hereafter designated the noncanonical Pntr site for HutC binding.

A comparison of the DNase I digestion patterns in response to varying HutC protein concentrations (Fig. 2C) and to urocanate (Fig. 2D) consistently indicates that HutC binds strongly to a 28 bp DNA region (highlighted in red), which contains the canonical Phut site with three half-sites. In contrast, interactions with a 40 bp region containing the noncanonical Pntr site (highlighted in blue) are weaker, and the protection is abolished by low concentrations of urocanate (Fig. 2D).

A regulatory model is proposed for the *hutF* promoter of SBW25, based on the HutC-P*_hutF_* interaction data presented above (Fig. S3). This model involves the formation of three stable oligomeric states—dimer, tetramer, and hexamer—on two binding sites (Phut and Pntr), with their assembly being regulated by urocanate concentration. In contrast, for PAO1, only the dimeric form is stably formed at the Phut site of *hutF* promoter.

### Role of the noncanonical HutC-targeting Pntr site in histidine catabolism

**Fig 3:**
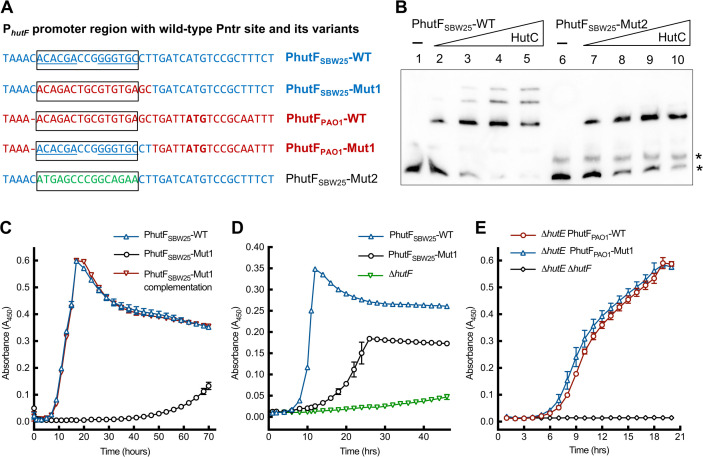
The HutC-binding Pntr site is functionally required for histidine utilization. (**A**) Alignment of P*_hutF_* sequences in the HutC protected region containing the Pntr site. PhutF_SBW25_-WT and PhutF_PAO1_-WT represent the wild-type alleles in strains SBW25 and PAO1, shown in blue and red fonts, respectively. PhutF_SBW25_-Mut1 and PhutF_PAO1_-Mut1 are mutants in which the boxed Pntr site region was reciprocally swapped between SBW25 and PAO1. PhutF_SBW25_-Mut2 is a Pntr site-eliminated variant used as a probe DNA in *in vitro* analyses of HutC-binding activities. (**B**) EMSA performed using His_6_-tagged HutC from SBW25 and P*_hutF_* probe DNA with and without the Pntr site. HutC was added at 0, 73, 219, 365, 584, 0, 73, 219, 365, and 584 nM in lanes 1–10, respectively. (**C**) Growth of wild-type SBW25 (PhutU_SBW25_-WT) and its derived Pntr site mutant MU59-86 (PhutF_SBW25_-Mut1) in M9 salt medium (MSM) supplemented with 10 mM histidine. Also included is a genetic complementation strain MU62-23 carrying wild-type *hutF* at the *att*Tn7 site. (**D**) Growth of wild-type SBW25 and its Pntr site mutant MU59-86 in MSM supplemented with succinate (5 mM) and histidine (1 mM). (**E**) Growth comparison of PAO1 ∆*hutE*-derived mutants carrying *hutF* with wild-type promoter (strain PBR1020) and with a promoter variant with Pntr site (strain MU63-95). A double deletion mutant (MU67-4) was included as a control. *P. aeruginosa* strains were grown in MSM supplemented with histidine (10 mM) as the sole carbon and nitrogen sources.

Data presented above identified a noncanonical Pntr site for HutC binding in the *hutF* promoter of *P. fluorescens* SBW25. Notably, this Pntr site is commonly found in strains of *P. fluorescens*, *Pseudomonas chlororaphis*, *Pseudomonas orientalis*, and *Pseudomonas simiae*, but it is absent in many other *Pseudomonas* species, such as *P. aeruginosa* (Fig. S4). To test the functionality of the Pntr site, we first performed an EMSA using a mutant probe, PhutFC-256, carrying a P*_hutF_* variant in which the Pntr site was replaced by a random sequence (PhutF_SBW25_-Mut2, Fig. 3A). As expected, elimination of the Pntr site resulted in a single shifted band in EMSA with HutC_SBW25_ (Fig. 3B). This further confirmed the specific HutC interactions with the Pntr site.

Next, we constructed two *Pseudomonas* mutants: MU59-86, derived from *P. fluorescens* SBW25 with its Pntr site being replaced by corresponding sequence in the *hutF* promoter of *P. aeruginosa* PAO1 (variant PhutF_SBW25_-Mut1, Fig. 3A); and conversely, a Pntr site was introduced into the *hutF* promoter of *P. aeruginosa* PAO1 (mutant MU63-95 with P*_hutF_* variant PhutF_PAO1_-Mut1, Fig. 3A). Considering that PAO1 possesses a branched pathway for FIGLU (Fig. 1A), the P*_hutF_* variant was constructed in the genetic background of PAO1Δ*hutE*, which grows normally on histidine through the HutFG branch of the histidine catabolic pathway (19).

As shown in Fig. 3C and D, when histidine was used as the sole carbon source, elimination of the Pntr site in *P. fluorescens* SBW25 caused severe growth defects (strain MU59-86), which were complemented by an extra copy of wild-type *hutF* integrated into the *att*Tn7 site (strain MU62-23). In contrast, PAO1 mutants (PBR1020 and MU63-95) with and without the Pntr site in P*_hutF_* promoter grew comparably on histidine, suggesting that the Pntr site is not functionally required for efficient histidine utilization in *P. aeruginosa*.

These findings led to the hypothesis that the SBW25 Pntr site plays an important role in the tight control of HutF expression, which is crucial for preventing the intracellular accumulation of FIGLU, a toxic intermediate of the *hut* pathway. Indeed, histidine became toxic for SBW25 mutant (MU59-86) with the elimination of the Pntr site in the *hutF* promoter (Fig. S5). This is evidenced by the growth of the mutant in M9 salt medium (MSM) supplemented with glutamate (5 mM) and varying concentrations of histidine. A histidine concentration-dependent inhibition was observed in the mutant (Fig. S5B).

#### *In silico* prediction of low-affinity HutC target sites in the PAO1 genome

Building on our understanding of HutC-mediated regulation of the *hut* operons, we next sought to identify potential HutC target sites beyond the *hut* locus at a genome-wide scale and to test the hypothesis that HutC functions as a direct global regulator of virulence-associated traits. To this end, a probability matrix was generated from the alignment of P*_hutU_* and P*_hutF_* promoter sequences from 12 *Pseudomonas* species and was then used for a genome-wide search of putative HutC targets in the genome of *P. aeruginosa* PAO1. This *in silico* analysis led to the identification of 172 candidates, including P*_hutU_* and P*_hutF_*, with a *P* value less than 10^−4^. A full list of the predicted HutC target sites is provided in Table S3, along with functional annotations of the neighboring genes. Intriguingly, a wide range of virulence-associated traits are under the potential control of HutC, including cell motility, biofilm formation, antibiotic resistance, pyoverdine (Pvd) synthesis, fatty acid catabolism, and nutrient acquisition.

Six Phut sites of our interest are summarized in Table 1. They were selected for further experimental verification mainly because they are linked to virulence traits, have the target site located within 200 bp upstream of the coding region, and display relatively high similarities to the well-characterized Phut site palindrome (TGTA-N_2_-TACA). More specifically, Arr is known as a regulator responsible for tobramycin-induced biofilm formation (27). The *faoA* gene is co-transcribed with *faoB*, encoding the alpha and beta subunits of the fatty acid oxidation complex, involved in the catabolism of long-chain fatty acids and required by *P. aeruginosa* PAO1 for surgical wound infection (28). The *cupA1* gene encodes the major structural component of the CupA fimbriae (pilus fibers), which promotes irreversible attachment to biotic and abiotic surfaces, an early essential step in biofilm formation (29). The divergently transcribed *cgrA* encodes a regulator essential for the phase-variable expression of *cupA* genes under anaerobic conditions (30). The sigma factor AlgU is a key regulator for robust biofilm formation (31, 32), while the divergently organized *nadB* gene encodes L-aspartate oxidase, an enzyme involved in NAD^+^ biosynthesis that potentially supports biofilm robustness by contributing to cellular energy production (33). PA1292 encodes a probable 3-mercaptopyruvate sulfurtransferase enzyme containing a Rhodanese-like domain, implicated in cyanide detoxification and potentially contributing to increased antibiotic resistance (34). The *bswR* gene and PA2781 are co-transcribed and associated with regulating swarming motility and biofilms, respectively (35).

**TABLE 1:** HutC target sites subjected to *in vitro* protein-DNA interaction assays

Site sequence^*a*^	Location^*b*^	Locus tag	Gene product	Function
TGCTTGTATTTTCAAC	←∙→	PA2819	Hypothetical protein	
		PA2818	Arr, aminoglycoside response regulator	Biofilm formation
TCCTTGTGCGTACAGG	→∙→	PA2780	BswR, bacterial swarming regulator	Cell motility
		PA2781	Hypothetical protein	
AGCTTGAACTTACACA	←∙→	PA3015	Hypothetical protein	
		PA3014	FaoA, fatty-acid oxidation complex alpha-subunit	Fatty acid catabolism
TAGTCGGAAATACAAG	←∙→	PA2128	CupA1, fimbrial subunit	Surface attachment
		PA2127	CgrA, cupA gene regulator A	Biofilm formation
TGCATGTCTGGACAAA	→∙→	PA1293	Hypothetical protein	
		PA1292	3-mercaptopyruvate sulfurtransferase	Sulfur metabolism and stress adaptation
CCCTAGTATATAGAAG	←∙→	PA0761	NadB, L-aspartate oxidase	NAD^+^ biosynthesis, energy metabolism
		PA0762	AlgU, alginate biosynthesis	Biofilm formation

^*a*^ Putative Phut half sites are underlined.

^*b*^ HutC-target site (filled circle) is shown together with genes located in the immediate vicinity of the *P. aeruginosa* PAO1 genome.

**Fig 4:**
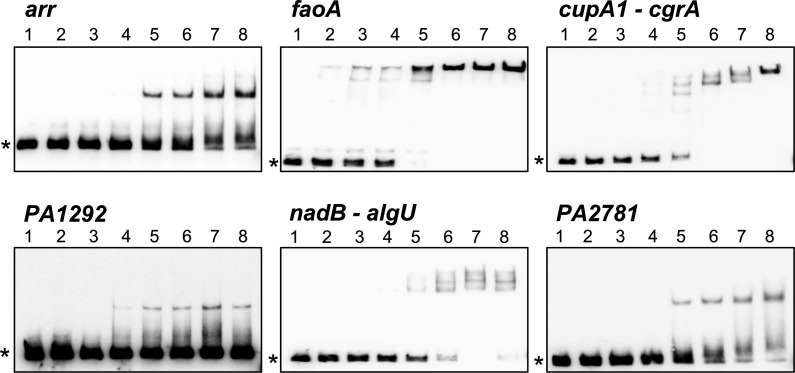
HutC binding to target sites beyond histidine utilization. Gel mobility shift assays were performed using His_6_-tagged HutC_PAO1_ added at increasing concentrations from lanes 2 to 8 (Table S1). No protein was included in lane 1 as a control. Asterisks at the left-hand side of images denote free probe DNAs.

To determine whether the HutC_PAO1_ protein can interact with the abovementioned target sites *in vitro*, EMSA was performed using a panel of six biotinylated DNA probes encompassing the putative Phut sites (Fig. 4). A protein concentration-dependent shift was generally observed for all six candidates. Moreover, the EMSA profiles enabled estimation of their binding affinities, except for the PA1292 promoter. As expected, the *K_d_* values ranged from 4.0 to 7.0 μM, which are higher than those of the P*_hutU_* and P*_hutF_* promoters (Table S1). The relatively lower binding affinities for non-*hut* promoters suggest that HutC exerts its global regulatory roles at high intracellular concentrations, when histidine (or urocanate) inducer is abundant in the environment, as HutC is transcribed under self-regulation.

### HutC contributes to tobramycin-induced biofilm formation

**Fig 5:**
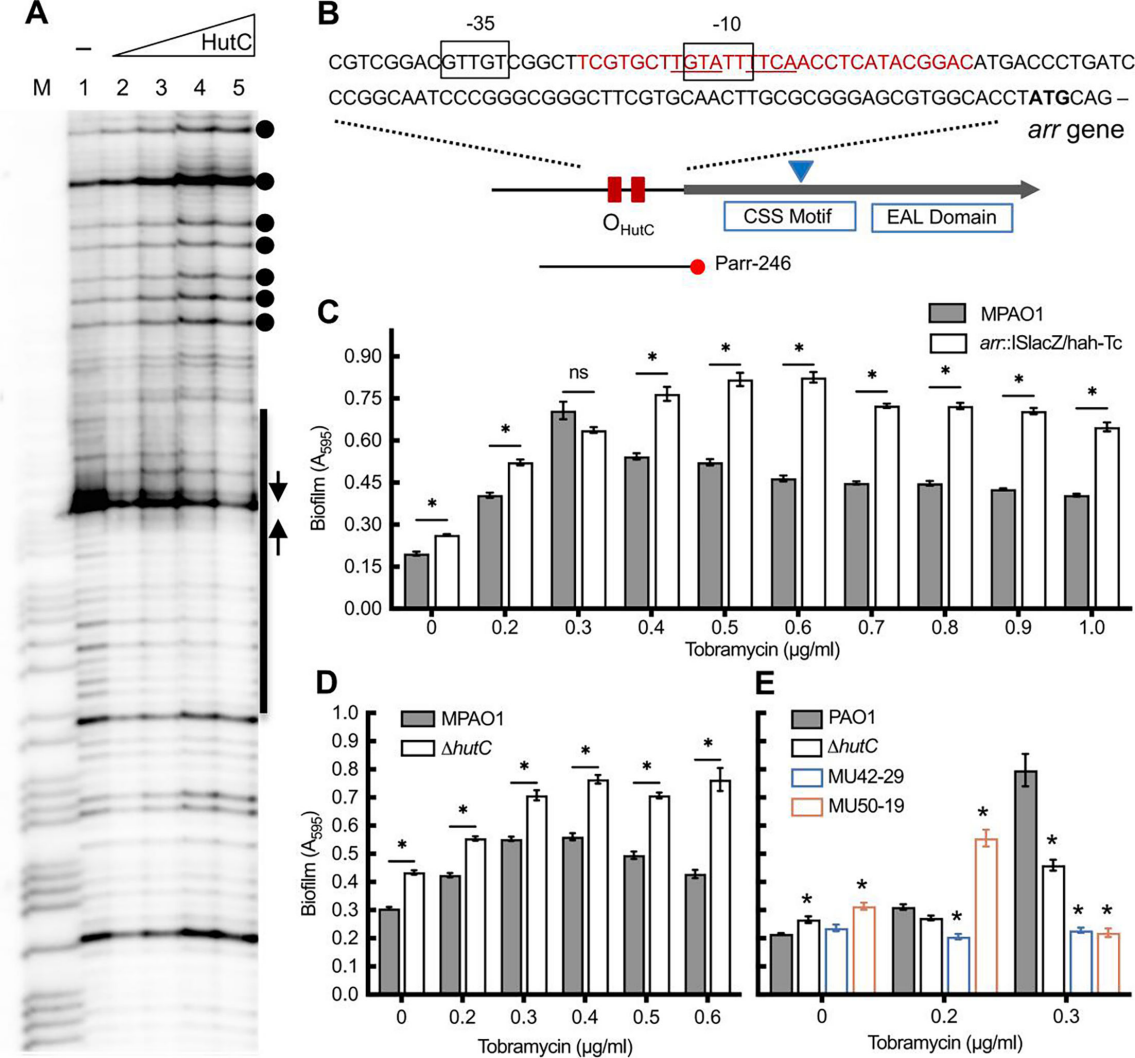
Involvement of HutC in Arr-mediated antibiotic-induced biofilm formation. (**A**) Determination of the HutC-binding site in *arr* promoter region. DNase I footprinting was performed using 3′ biotin-labeled probe Parr-246 and His_6_-tagged HutC_PAO1_ added at increasing concentrations (0, 1.0, 2.0, 3.0, and 5.0 µM) in lanes 1–5. M, G+A marker. The HutC-protected region, Phut sites, and hypersensitive DNase I cleavage sites are shown in black bar, inverted arrows, and filled circles, respectively. (**B**) Genetic map of PAO1 *arr* locus. The HutC-protected region is shown in red font, and the inverted triangle denotes the insertion position of ISlacZ/hah-Tc in CSS motif of *arr* gene product in mutant MU61-33. (**C**) Assessment of biofilm formation LB by wild-type MPAO1 and its derived *arr* inactive mutant MU61-33. (**D**) Biofilm formed by MPAO1 and an isogenic ∆*hutC* mutant in LB. (**E**) Biofilms formed by PAO1, ∆*hutC*, and their derived MU42-29 for *hutC* complementation (∆*hutC att*Tn7::*hutC*), and mutant MU50-19 for *hutC* over-expression (PAO1 pME6010-*hutC*) in MHB medium. Biofilms (*A_595_*) were quantified 8 h after inoculation, and the data are means and standard errors of eight replicate cultures. Asterisk denotes a significant difference above the bar between wild type and mutant tested in parallel with the same tobramycin concentration at *P* < 0.05.

To investigate the phenotypic consequences of *hutC* inactivation, an in-frame deletion mutant was additionally constructed in the genetic background of *P. aeruginosa* MPAO1, a wild-type variant of the transposon (Tn) mutant library at the University of Washington (36, 37). Inactivation of *hutC* led to constitutive expression of the *hut* operons, as confirmed using an integrated P*_hutU_*-*lacZ* fusion at the *att*Tn7 site (Fig. S6). Considering that a large proportion of HutC target genes is linked to biofilm formation, our functional analysis was focused on the *arr* gene, which encodes a key regulator of tobramycin-induced biofilms. First, we obtained further evidence that urocanate can dissociate the HutC/P*_arr_* interaction *in vitro*. In an EMSA using His_6_-tagged HutC_PAO1_ and a biotinylated probe DNA containing the P*_arr_* promoter region, a decrease in shifted bands was clearly coupled with an increase in free probe DNAs (Fig. S7). Next, a DNase I footprinting analysis was performed to determine the specific HutC-binding site (Fig. 5A). Results showed that a 30 bp P*_arr_* region was protected (Fig. 5B), which overlaps a putative σ^70^ promoter predicted by the SAPPHIRE program (38) and contains the predicted Phut site for HutC binding. The presence of hypersensitive bands further supports specific protein-DNA interaction (Fig. 5A).

Armed with new knowledge that HutC specifically targets the *arr* promoter, we next conducted a biofilm assay using strain MPAO1 grown in lysogeny broth (LB) medium. The results generally confirmed the previously reported role of tobramycin in inducing biofilm formation when added at sub-inhibitory concentrations ≤1.0 μg/mL (Fig. S8). However, inactivation of either *arr* or *hutC* led to a significant increase in biofilm formation, both in the presence and absence of tobramycin (Fig. 5C and D). Similar results were obtained with another *arr* Tn mutant MU61-32 (*arr*::ISphoA/hah-Tc; Fig. S9).

To further examine the role of *hutC* in biofilm induction, PAO1 and its derived Δ*hutC* mutant were analyzed in Mueller-Hinton broth (MHB), a standard medium for antimicrobial susceptibility testing. Also included were strain MU42-29 (∆*hutC*, *att*Tn7::*hutC*), representing *hutC* complementation at a separate *att*Tn7 site, and strain MU50-19 (PAO1 carrying pME6010-*hutC*), in which *hutC* is overexpressed under the control of a strong constitutive promoter (39). Intriguingly, OE of *hutC* led to a severe defect in planktonic growth but enhanced biofilm formation, particularly in the presence of 0.2 μg/mL tobramycin (Fig. 5E ; Fig. S10). The complementation strain did not consistently reproduce the wild-type phenotype, suggesting that tight regulation of *hutC* expression is critical. It appears that HutC plays a complex regulatory role in biofilm formation that cannot be explained solely by its direct regulation of the *arr* gene. Indeed, a biofilm screen of eight Tn mutants potentially regulated by HutC revealed significant alterations in most, except for the mutant of PA2359 (Fig. S11). Collectively, these data implicate an important regulatory direct role for HutC in biofilm formation, including the induction in response to tobramycin.

### Phenotypic consequences of *hutC* deletion on Pvd, motility, and virulence

Pvd is the main siderophore produced by *P. aeruginosa* for iron acquisition and is a critical determinant of its pathogenicity (40, 41). A HutC-binding site is present in the promoter regions of *fpvA* and *fpvB*, which encode receptor proteins for the uptake of the Pvd-Fe^3+^ complex. Interestingly, the ferric uptake regulator (*fur*) gene also contains a putative Phut site near its promoter region (Table S3). Fur is the master regulator of cellular iron metabolism in *P. aeruginosa*, acting as the primary transcriptional repressor of Pvd synthesis under iron-replete conditions.

**Fig 6:**
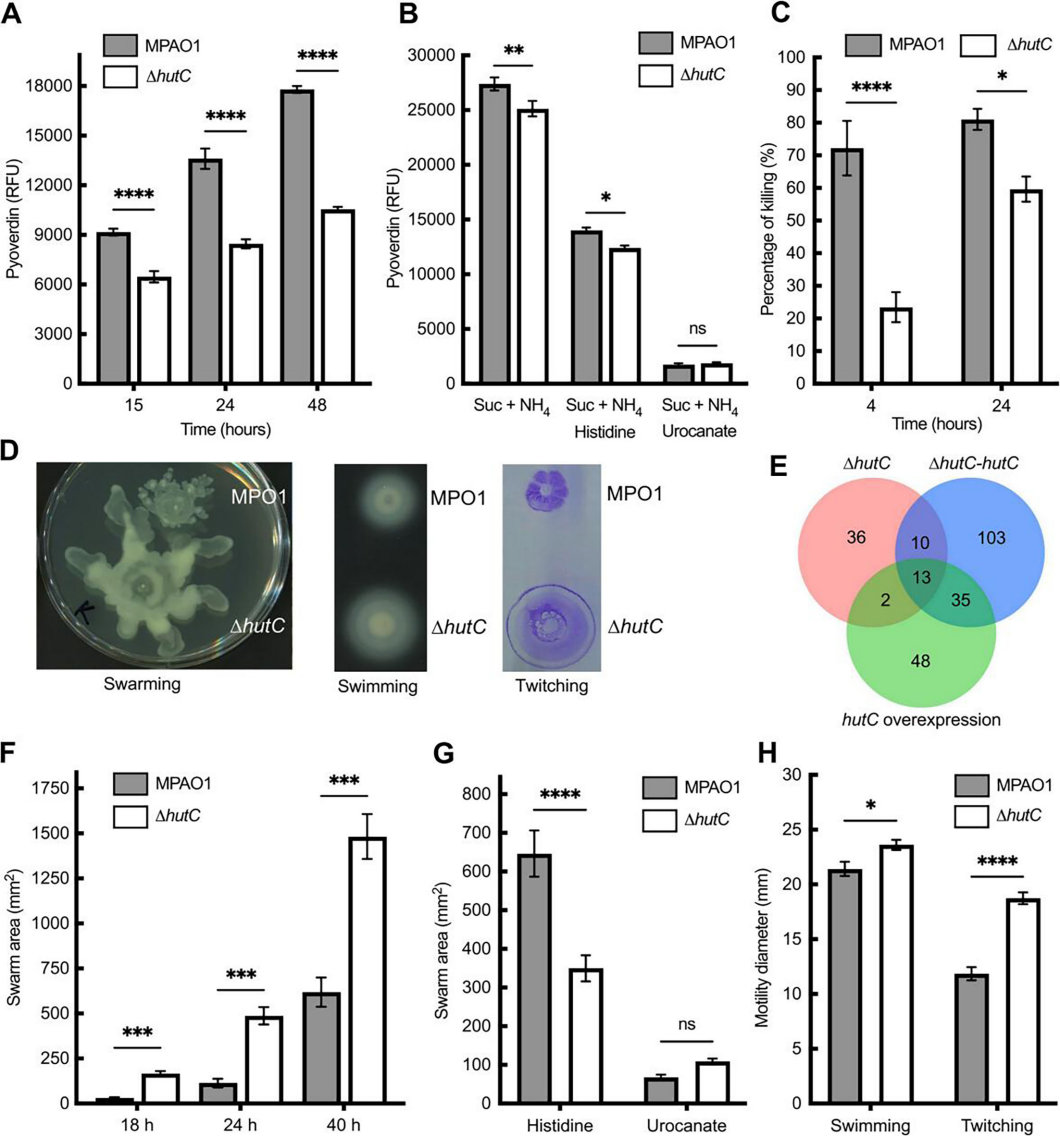
Consequences of *hutC* inactivation on Pvd production, motility, and virulence. (**A**) Pvd production in KB medium at 15, 24, and 48 h after inoculation. (**B**) Effects of histidine or urocanate (10 mM) on Pvd production in “MSM + succinate + NH_4_Cl.” (**C**) Virulence on the *Caenorhabditis elegans* infection model. (**D**) Representative images of the swarming, swimming, and twitching assays. (**E**) A Venn diagram showing the number of DEGs in three *hutC* mutants. MU57-46 (∆*hutC*), MU42-29 carrying *hutC* at *att*Tn7 site (∆*hutC-hutC*), and MU50-19 with *hutC* OE (PAO1 pME6010-*hutC*). (**F**) Swarming motility assayed on 0.5% nutrient broth agar containing 28 mM glucose. (**G**) Histidine or urocanate (5 mM) was added to the swarming medium (**F**), and swarm areas were measured 40 h after inoculation. (**H**) Swimming and twitching motilities assayed on 0.25% and 1.0% LB agar, respectively. Data are means and standard errors of >4 replicate plates for virulence and >6 repeats for other assays. Asterisks denote significant difference between MPAO1 and its derived ∆*hutC* mutant tested in parallel at *P* < 0.05 (*), 0.01 (**), 0.001 (***), and 0.0001 (****).

To investigate the role of *hutC* in Pvd regulation, we leveraged the intrinsic fluorescence of Pvd to quantify its production in MPAO1 and its derived mutant MU61-68 (Δ*hutC*), grown in nutrient-rich King’s B (KB) medium and in minimal MSM supplemented with histidine and urocanate. The results revealed that *hutC* inactivation caused a significant reduction of Pvd synthesis in both KB and the minimal medium supplemented with succinate and ammonium as the carbon and nitrogen sources (Fig. 6A and B). While histidine inhibited Pvd synthesis, urocanate exerted a more pronounced inhibitory effect (Fig. 6B). As shown in Fig. S12, urocanate inhibited Pvd synthesis in a concentration-dependent manner. Together, these findings consistently indicate an inhibitory role for histidine and urocanate in Pvd biosynthesis, potentially mediated by HutC.

Next, cell motility was assessed through swarming, swimming, and twitching assays with MPAO1 and its derived Δ*hutC* mutant (MU61-68). On 0.5% nutrient broth agar supplemented with glucose, Δ*hutC* exhibited a hyper-swarming phenotype, with a swarming area more than twice that of MPAO1 (Fig. 6D and F). The addition of histidine had no effect on the swarming behaviors of MPAO1; however, it significantly reduced the swarming of the Δ*hutC* mutant (Fig. 6G). Notably, swarming was abolished by the presence of urocanate (Fig. 6G). Results of swimming and twitching assays revealed a significant increase for Δ*hutC* relative to wild type (Fig. 6D and H). In the genetic background of PAO1, deletion of *hutC* also resulted in a super-swarming phenotype (Fig. S13A). Interestingly, the addition of histidine changed the way of Δ*hutC* spreading but not for wild-type PAO1 (Fig. S13B), and the super-swarming phenotype of Δ*hutC* was abolished when *hutC* was over-expressed under the control of constitutive promoter Pk in plasmid pME6010 (Fig S13C). Together, the data indicate that HutC plays a significant regulatory role in motility, particularly swarming in both MPAO1 and PAO1.

To determine the virulence effects of *hutC* inactivation, a fast-killing assay was conducted with *Caenorhabditis elegans* grown at the L4 stage (42). Worms were transferred onto peptone-glucose-salts (PGS) agar plates containing a bacterial lawn of either MPAO1 or its derived Δ*hutC* mutant. A non-pathogenic *Escherichia coli* HB101 strain was used as a negative control. The percentages of worms killed after exposure to the bacterial treatments are plotted in Fig. 6C. As expected, all worms survived exposure to *E. coli* over the course of 24 h. At 4 h post-exposure, MPAO1 was able to kill 72.2% of the worms, whereas a death rate of 59.6% was observed for the Δ*hutC* mutant. The difference remained significant after 24 h of exposure. Thus, the nematode assay data support the involvement of HutC in the regulation of *P. aeruginosa* virulence.

### Effects of *hutC* genetic alteration on global gene expression

Finally, to obtain a glimpse of the global regulatory roles of *hutC*, transcriptome sequencing (RNA-seq) was performed using wild-type PAO1 and three isogenic *hutC* mutants: MU57-46 (Δ*hutC*), MU42-29 (Δ*hutC* with *hutC* complementation at the *att*Tn7 site), and the *hutC* constant OE strain MU50-19. Results are summarized in Fig. 6E and Fig. S14. Total RNA was prepared from three biological replicates of exponentially growing cells cultured in “MSM + histidine.” Under this condition, the *hut* operon is fully induced; however, deletion of the *hutC* repressor is expected to further increase *hut* expression (Fig. S6). Consistent with this prediction, expression of the *hut* operons was upregulated in the Δ*hutC* mutant, whereas no significant difference was observed in the other two mutants, MU42-29 and MU50-19, both of which carry a functional copy of *hutC*. Interestingly, expression of denitrification genes in the nitrite/nitric oxide reductases cluster (PA0509–PA0527) and the nitrous oxide reductase cluster (PA3391–PA3396) showed a similar pattern as *hut*: both clusters were upregulated in mutants MU42-29 and MU50-19. Denitrification is recognized as an important virulence trait, providing energy to support anaerobic growth (43). Among the 247 differentially expressed genes (DEGs), genes involved in Pvd production and motility were identified, consistent with the phenotypes described above. Notably, the “complementation” strain MU42-29 (Δ*hutC* with *hutC* at *att*Tn7 site) exhibited the largest number of DEGs relative to the other two mutants, which either lacked *hutC* entirely (MU57-46) or constitutively overexpressed *hutC* (MU50-19). This observation suggests that the native genetic overlap between *hutF* and *hutC* promoters is critical for HutC-mediated autoregulation, enabling precise sensing of intracellular histidine level.

## DISCUSSION

**Fig 7:**
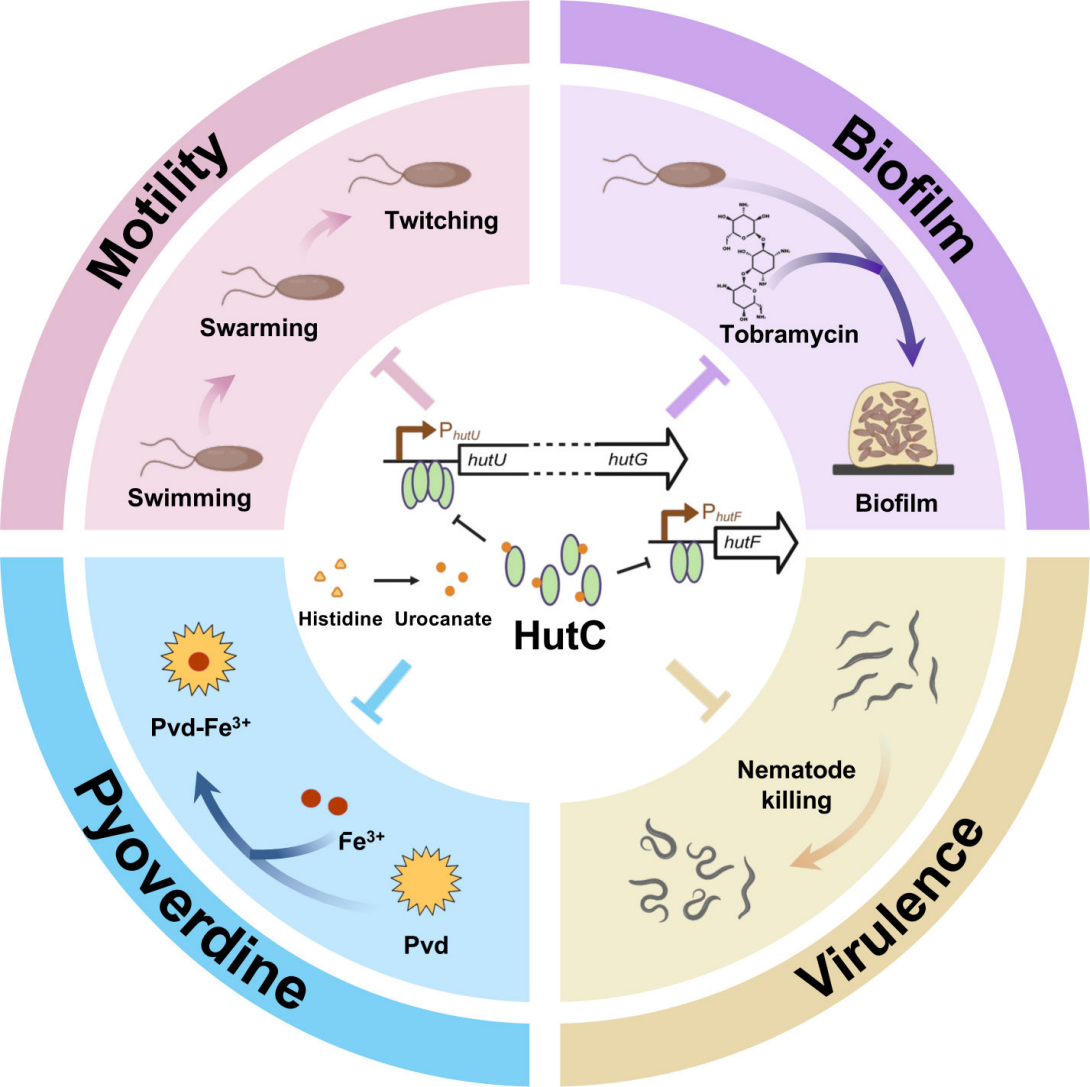
Outline of HutC-mediated gene regulation in *P. aeruginosa*. HutC represses *hut* gene expression by forming a tetramer at the P*_hutU_* promoter and a dimer at the P*_hutF_* promoter, and the protein-DNA interactions are regulated by urocanate in a concentration-dependent manner. More importantly, HutC binds with lower affinity to at least 170 additional promoters in the PAO1 genome, many of which are associated with virulence. Deletion of *hutC* leads to a significant reduction in virulence, impaired Pvd production, altered motility, and increased tobramycin-induced biofilm formation.

Here, we describe the genetic and biochemical characterization of HutC in *Pseudomonas*, focusing on its regulatory roles in the expression of *hut* genes and genes beyond histidine catabolism in *P. aeruginosa* (outlined in Fig. 7). Our analysis led to two key findings: (i) a noncanonical HutC target site (Pntr) is present in the *hutF* promoter of certain *Pseudomonas* strains but absent in *P. aeruginosa*; and (ii) HutC exhibits low-affinity binding to the promoters of virulence-related genes and contributes to the expression of virulence traits, including antibiotic-induced biofilm formation, Pvd production, and cell motility. Collectively, our data highlight the functional significance of low-affinity DNA-binding by TFs in gene regulation and suggest that HutC may represent a potential target for developing new strategies to combat *P. aeruginosa* infections.

In addition to the canonical Phut site, HutC is known to bind a distinct Pntr site, which is also targeted by NtrC for autoregulation of the *ntrB-ntrC* operon in *P. fluorescens* SBW25 (22). The NtrBC two-component system is a master regulator of nitrogen metabolism in *Pseudomonas* (16, 44). Under nitrogen-starved conditions, NtrC activates its own expression as well as that of various catabolic genes, including the *hutU* operon. Competitive binding between HutC and NtrC at the *ntrBC* promoter plays a key role in controlling the histidine catabolic rate (22), ensuring that SBW25 cells maintain a nitrogen-starved physiological state during histidine degradation, a process that yields more nitrogen than carbon (22).

The noncanonical Pntr site identified in this work represents a second compelling example demonstrating that HutC can specifically target DNA with little sequence similarity to the canonical Phut motif. While EMSA and DNase I footprinting, combined with site-directed mutagenesis, provide strong supporting evidence, the molecular basis of this binding specificity requires further biochemical investigation. Nevertheless, our finding adds to the growing body of evidence challenging the classic view of “one TF, one motif” and introduces a prokaryotic TF (i.e., HutC) into the timely reconsideration of the noncanonical TF-binding specificity (45–47). Proposed mechanisms include recognition and binding based on DNA shape readout, which emphasizes the role of overall DNA structure rather than sequence alone (45, 48).

Regarding the biological functions of the noncanonical Pntr site, *hutF* is located separately from the other structural genes involved in histidine utilization in *Pseudomonas*, and thus, a specialized regulatory mechanism may have evolved to prevent intracellular accumulation of the toxic intermediate FIGLU (Fig. 1A). Indeed, deletion of the Pntr site in SBW25 led to significant growth defects on histidine. However, no such Pntr site is present in the *hutF* promoter of *P. aeruginosa*, which may be partially explained by the presence of an alternative pathway for the enzymatic breakdown of FIGLU (Fig. 1A, HutE). The *hutE* gene is co-transcribed with a FIGLU-inducible IcIR-type regulator, HutR, both of which are likely horizontally acquired in *P. aeruginosa* (19). Their complex impacts on HutC-mediated *hut* gene expression will be reported separately.

Urocanate has been hypothesized to serve as a signaling molecule for bacterial host perception, mainly due to its tissue-specific accumulation and the emerging global regulatory role of HutC beyond histidine catabolism (6). Urocanate was first identified in the urine of a dog, hence the name. In addition to the skin, urocanate is also found in the liver and brain of both healthy and diseased individuals. Its levels in skin, urine, and fecal samples serve as metabolic biomarkers for a range of diseases (13). However, in the absence of comprehensive, purpose-designed analyses, it is possible that histidine itself also contributes to host-specific signaling. Histidine can exert similar effects on HutC-mediated gene regulation following its intracellular conversion to urocanate (Fig. 1A). *P. aeruginosa* strains possess transporters specific for both histidine (HutTh, PA5097) and urocanate (HutTu, PA5099) (49). In PAO1, Tn insertion upstream of *hutTu* resulted in a significant reduction in T3SS-mediated cytotoxicity, where the expression of exoenzyme S was abolished, and the mutant was unable to lyse macrophages (17). The data presented here suggest that the virulence defects caused by Tn insertion in the *hut* operon are likely mediated by the regulatory effects of HutC in response to histidine or urocanate.

A total of 172 HutC target sites were identified by *in silico* analysis of the PAO1 genome, showing varying degrees of sequence similarity to the canonical Phut site. However, the actual number of HutC targets is likely higher when homologs of the noncanonical Pntr sites are also considered. Many of the HutC targeting loci are associated with bacterial pathogenesis. Accordingly, inactivation of *hutC* resulted in a significant reduction in virulence in the *C. elegans* infection model. Consistent with our expectation, the *hutC* deletion mutant exhibited impaired Pvd production and altered cell motility, including swimming, swarming, and twitching. The related genes were differentially expressed in *hutC* mutants as revealed by transcriptome analysis using bacterial cells grown on histidine as the sole source of carbon and nitrogen. Notably, *hutC* was identified in a Tn insertion assay for mutants defective in swarming motility in *P. aeruginosa* PA14 (50). The regulatory role of *hutC* in virulence has also been observed in non-*Pseudomonas* pathogens such as *Brucella abortus* (51), *Acinetobacter baumannii* (52), and *Vibrio parahaemolyticus* (53).

Finally, we obtained strong evidence that HutC specifically interacts with the promoter region of the *arr* gene, which encodes a c-di-GMP phosphodiesterase involved in tobramycin-induced biofilm formation (27). The Δ*arr* mutant exhibited stronger biofilm induction than the wild-type strain MPAO1 (Fig. 5). This result is consistent with the expected role of c-di-GMP phosphodiesterase in lowering the intracellular pool of c-di-GMP, as high levels of intracellular c-di-GMP enhance biofilm formation, whereas low levels facilitate biofilm dispersion (54). Interestingly, *hutC* deletion also led to increased biofilm induction in MPAO1, but this effect was not fully observed in strain PAO1. HutC likely modulates biofilm induction not only through *arr* but also via other unidentified pathways that warrant further investigation. Together, our data suggest that targeting host perception mechanisms mediated by nutrition-specific regulators such as HutC could contribute to the development of new therapeutic strategies to combat the growing threat of antibiotic resistance.

## MATERIALS AND METHODS

### Bacterial strains and growth conditions

A summary of *Pseudomonas* strains and plasmids used in this study is provided in Table 2. Bacteria were routinely grown in LB medium at 37°C for *P. aeruginosa* and *E. coli* and at 28°C for *P. fluorescens* SBW25. When grown in minimal MSM-based medium, each carbon substrate was supplemented at 10 mM unless otherwise indicated (20). Succinate, histidine, and urocanate were purchased from Sigma-Aldrich (Merck KGaA, Darmstadt, Germany). To ensure physiological comparability among *Pseudomonas* strains, inoculants were prepared by centrifuging cells grown in LB and resuspending them in an equal volume of MSM lacking carbon and nitrogen sources. After washing twice, bacterial cells were then subjected to starvation in MSM for 2 h prior to inoculation into the tested medium. Growth curves were obtained in 96-well plates as previously described, using a Synergy 2 plate reader equipped with Gen5 software (BioTek Instrument) (21). Absorbance data (*A_450_* or *A_600_*) were normally collected at 10 min intervals, although data presented are plotted at hourly or 2-hourly intervals for clarity.

**TABLE 2:** Bacterial strains and plasmids used in this study

Strain or plasmid	Genotype and characteristics	Source or reference
*P. fluorescens* strains		
SBW25	Wild-type strain isolated from phyllosphere of sugar beet	18
MU35-86	Δ*hutF*, SBW25 devoid of Pflu0358	This work
MU59-86	SBW25 carrying *hutF* promoter variant PhutF_SBW25_-Mut1	This work
MU62-23	Strain MU59-86 carrying SBW25 *hutF* gene at the *att*Tn7 site	This work
*P. aeruginosa* strains		
PAO1	Wild type, laboratory stock	Lab stock
PBR1020	Δ*hutE*, PAO1 with the deletion of PA3175	19
MU63-95	Δ*hutE*, PAO1 carrying the *hutF* promoter variant PhutF_PAO1_-Mut1	This work
UOA67-4	Δ*hutE* Δ*hutF*, PAO1 with in-frame deletion of PA3175 and PA5106	This work
MPAO1	Wild-type strain from the distributor of the MPAO1 Tn mutant library at the University of Washington, Seattle	37, 55
MU61-32	*arr*::ISphoA/hah-Tc, MPAO1 carrying Tn insertion at the 688 nt position of PA2818 (1,578 nt in total), strain PW5719	55
MU61-33	*arr*::ISlacZ/hah-Tc, MPAO1 carrying Tn insertion at the 672 nt position of PA2818 (1,578 nt in total), strain PW5718	55
MU61-71	Δ*arr*, MPAO1 devoid of PA2818	This work
MU61-68	Δ*hutC*, MPAO1 devoid of PA5105	This work
MU57-46	PAO1 Δ*hutC*, deletion of PA5105	This work
MU42-29	PAO1 Δ*hutC* containing PAO1 *hutC* gene at *att*Tn7 site for *hutC* complementation	This work
MU50-19	PAO1 containing pME6010-*hutC* for *hutC* over-expression, Tc^R^	This work
Plasmids		
pRK2013	Helper plasmid, Tra^+^, Km^R^	56
pUIC3	Integration vector with promoterless *lacZ*, Mob^+^, Tc^R^	57
pUIC3-133	pUIC3 containing 900 bp DNA fragment for *hutF* deletion in SBW25	This work
pCR8/GW/TOPO	Cloning vector, Sp^R^	Invitrogen
pTrc99A-*hutC*	pTrc99A recombinant plasmid for the expression of His_6_-tagged HutC from *P. fluorescens* SBW25, Ap^R^	21
pET14b-*hutC*	pET14b recombinant plasmid for the expression of His_6_-tagged HutC from *P. aeruginosa* PAO1, Ap^R^	Lab stock
pUX-BF13	Helper plasmid for transposition of mini-Tn7 element, Ap^R^	58
pUC18-mini-Tn7T-Gm-GW	Gateway destination vector for gene insertion at the single *att*Tn7 site in the chromosome, Ap^R^, Gm^R^	59
pUC18-mini-Tn7T-LAC	Mini-Tn7 vector for gene complementation, Ap^R^, Gm^R^	59
pUC18-mini-Tn7T-GM-lacZ	Mini-Tn7 vector for transcriptional fusion to promoterless *lacZ*, Ap^R^, Gm^R^	59
pUC18-P*_hutU_-lacZ*	pUC18-mini-Tn7T-GM-lacZ containing *lacZ* fusion to P*_hutU_* from PAO1, Ap^R^, Gm^R^	This work
pUC18-*hutC*	pUC18-mini-Tn7T-LAC carrying *hutC* of *P. aeruginosa* PAO1, Ap^R^, Gm^R^	This work
pME6010-*hutC*	pME6010 carrying *hutC* of *P. aeruginosa* PAO1, Tc^R^	This work
pUC18-*hutF*	pUC18-mini-Tn7T-Gm-GW carrying *hutF* from *P. fluorescens* SBW25 with its original promoter PhutF_SBW25_-WT	This work
pUC18-mini-Tn7T-*hutF*-mut1	pUC18-mini-Tn7T-Gm-GW carrying *hutF* from *P. fluorescence* SBW25 with promoter variant PhutF_SBW25_-Mut1	This work

### Strain construction

Standard protocols were used for the isolation of plasmid DNAs, restriction endonuclease digestion, ligation, and PCR. PCRs were performed using *Taq* DNA polymerase purchased from Invitrogen Ltd. (Auckland, New Zealand). Oligonucleotide primers were synthesized by Integrated DNA Technologies Inc. (Singapore) and are shown in Table S2. Site-directed mutagenesis was performed using the established protocol of gene splicing by overlap extension PCR, combined with a two-step allelic exchange strategy employing the suicide-integration vector pUIC3 (20, 57). Briefly, two similarly sized DNA fragments flanking the mutated site were amplified using two pairs of oligonucleotide primers and then joined in a third PCR via complementary sequences at their internal ends. The resulting PCR product was first cloned to the pCR8/GW/TOPO (Invitrogen) vector, and sequence identity was verified by Sanger’s DNA sequencing (Macrogen Inc., Seoul, South Korea). Next, the DNA fragment was sub-cloned into pUIC3 at the *Bgl*II site and introduced into *Pseudomonas* by tri-parental mating with the help of pRK2023. Integration of pUIC3 into the genome via a single homologous recombination event of the cloned DNA was selected on LB agar plates supplemented with tetracycline (Tc, 15 μg/mL), nitrofurantoin (Nf, 100 μg/mL; to counter-select *E. coli*), and 5-bromo-4-chloro-3-indolyl-β-D-galactopyranoside (X-Gal, 60 μg/mL). Double crossover mutants that had lost the chromosomally integrated pUIC3 vector were selected using a D-cycloserine (800 μg/mL) enrichment process, as previously described for *P. fluorescens* SBW25. When applied to *P. aeruginosa* PAO1, the concentration of D-cycloserine was increased to 1,100 μg/mL to ensure effective elimination of plasmid-bearing, Tc-resistant cells. Finally, the resulting white Tc-sensitive colonies were subjected to PCR analysis to distinguish the desired mutant from wild-type revertants.

Complementation of the *hutF* and *hutC* genes was performed by cloning the PCR-amplified DNA into vector pUC18-mini-Tn7T-Gm-GW and pUC18-mini-Tn7T-LAC, respectively (59). The resulting recombinant plasmids were then introduced into *Pseudomonas* genome by electroporation with the help of pUX-BF13, which carries transpose genes for integration of the cloned DNA fragment into a unique *att*Tn7 site in the genome. Transformants were selected on LB plates supplemented with gentamycin (20 μg/mL), and chromosomal integration downstream of the *glmS* gene was confirmed by PCR using primers SBW25-glmS/Tn7R109 and PAO1-glmS/Tn7R109 for *P. fluorescens* and *P. aeruginosa*, respectively (Table S2).

### EMSA and DNase I footprinting

The coding regions of HutC_SBW25_ and HutC_PAO1_ were PCR-amplified and cloned into the protein expression vectors pTrc99A and pET14a, respectively. Following sequence verification, the resulting recombinant plasmids were transformed into *E. coli* BL21(DE3) (60), and protein expression was induced by the addition of 1 mM isopropyl β-D-1-thiogalactopyranoside at mid-log phase (*A_600_*, ~0.6). The His_6_-tagged proteins were then purified using TALON metal affinity resin (Clontech Laboratories Inc.) following the manufacturer’s recommendation. Probe DNAs were biotin-labeled at the 5′ ends through PCR from genomic DNA with a biotinylated primer.

Protein-DNA interactions were carried out in a 20 μL reaction (pH 7.5) containing 10 mM HEPES, 5 mM MgCl_2_, 50 mM KCl, 1 mM dithiothreitol, and 1 µg of salmon sperm DNA. Probe DNA was added at a final concentration of 20 nM unless otherwise specified. After a 30 min incubation at room temperature, the samples were electrophoresed at ~4°C on a 5% polyacrylamide gel at 120 v in half strength Tris-borate-EDTA (TBE) buffer. Next, DNAs in the gel were electrophoretically transferred to a nitrocellulose membrane (Sigma-Aldrich) and fixed by oven baking at 80°C for 30 min. The immobilized biotin-labeled DNAs on the membranes were detected using the Chemiluminescent Nucleic Acid Detection Kit (Thermo Fisher Scientific) as per the manufacturer’s instructions. Images were captured using a LAS-4000 luminescent imager equipped with ImageQuant LAS 4000 software (Fujifilm) and further analyzed using ImageJ program. To determine the dissociation constant, the fraction of protein-DNA complex was plotted against protein concentration using a non-linear regression model (specific binding with Hill slope) in GraphPad Prism v7. *K_d_* was estimated from the curve as the protein concentration required to bind 50% of the probe DNA.

For DNase I analysis, the initial step of protein-DNA interaction was conducted under the same conditions as EMSA, but in a 50 μL reaction with 2 μM probe. The reaction was then mixed with 50 μL co-factor solution (5 mM CaCl_2_ and 10 mM MgCl_2_) prior to treatment with 0.02 units of DNase I (Invitrogen) for 6 min at room temperature. Digestion was terminated by 100 μL stop buffer containing 200 mM NaCl, 20 mM EDTA (pH 8.0), and 1% SDS. The digested DNA fragments were subjected to 1:1 phenol:chloroform treatment followed by ethanol precipitation at −80°C for 1 h. The resulting DNA pellets were air dried and resuspended in 8 μL of loading buffer composed of 95% (vol/vol) formamide, 0.05% (wt/vol) bromophenol blue, and 20 mM EDTA. After denaturing at 90°C for 15 min, samples were loaded to a 6% urea-polyacrylamide gel (21 × 40 cm) for electrophoresis in 1× TBE buffer, using the Sequi-Gen GT system (Bio-Rad Laboratories Pty). Probe DNAs were detected as described above for EMSA. A G+A marker synthesized from a chemical sequencing reaction was included in the assay to assist in determining protein-protected site.

### Phenotypic and virulence assays

Biofilm formation was quantitatively assayed in a 96-well microtiter plate (Greiner Bio-One), using the standard method of crystal violet staining as previously described (61, 62). Protocols for motility assays were adapted from previous studies with minor amendments (63, 64). Swimming was examined in 0.25% LB agar, whereas swarming was assayed on 0.5% nutrient broth (Difco) agar (Oxoid) supplemented with 28 mM glucose. Swimming and swarming plates were inoculated by gentle stabbing into the agar with a sharp, pointed plastic needle, using cells grown on 0.3% soft LB agar. Twitching was examined in 1% LB plate inoculated by stabbing agar all the way down to the agar-plastic interface. Plates were incubated facedown at 37°C for 48 h, followed by an additional 24 h at room temperature. To visualize the twitching zone, agar was carefully removed with a scalpel, and the plates were subjected to 0.1% crystal violet staining. Motilities were scored quantitatively by measuring bacterial expanding areas using the ImageJ program (65).

The ability to produce Pvd was estimated by measuring fluorescence of the supernatant at 460 nm with an excitation wavelength of 360 nm in a Synergy 2 multimode microplate reader (BioTek Instruments). Data are presented as relative fluorescence units normalized by absorbance at 600 nm (*A_600_*).

Virulence assay was conducted using the protocol described by Tan et al. (42), with slight modifications. Briefly, 30 mL overnight cultures of *P. aeruginosa* PAO1 and *E. coli* HB101 grown in LB were inoculated by spreading them over the entire surface of a 30 mm PGS agar plate (1% Bacto-Peptone, 1% NaCl, 1% glucose, 0.15 M sorbitol, and 1.7% agar). After a 16 h incubation at 37°C, the plates with bacterial lawns were cooled to room temperature, and each was seeded with 10 L4-stage *C. elegans* worms, which were maintained on NGM medium fed by *E. coli* HB101 (66). Both live and dead worms were counted after incubation at 25°C for 4 and 24 h. Worms that did not respond to touch for ~15 s were counted as dead, while those missing (presumably hiding at the edges of the plates) were excluded from the analysis.

### Transcriptome sequencing

PAO1 and its three isogenic *hutC* mutants were grown in triplicate in MSM + histidine (10 mM), and total RNA was extracted from cells at mid-log phase for RNA-seq analysis performed by Novogene Technology Co. Ltd. (Beijing, China) following their standard protocols. RNA integrity was assessed using an Agilent 2100 Bioanalyzer (Agilent Technologies, Inc.), and sequencing was conducted on an Illumina HiSeq 4000 platform. After removal of adapter sequences, rRNA reads, and low-quality reads, the clean reads were mapped to the reference genome (NC_002516.2) using Bowtie2 (67) and assembled using Rockhopper software (68). Gene expression levels were normalized using reads per kilobase of transcript per million mapped reads. DEGs were identified using edgeR with a false discovery rate <0.05 and an absolute log_2_ fold-change (|log_2_FC|) >1. Enrichment of Kyoto Encyclopedia of Genes and Genomes pathways was evaluated using *P*adj < 0.05 as the significance cutoff.

### *In silico* analysis and statistics

To determine putative HutC target sites in PAO1 genome, a probability matrix representing the HutC-binding palindromic motif (i.e., the canonical Phut site) was generated using the MEME motif discovery program (69). A 64 bp DNA region from the *hutF* and *hutU* promoters of 40 *Pseudomonas* strains, representing 12 species, was used as input. The conserved motif and its corresponding probability matrix were derived using the following parameters: motif discovery mode, classic; site distribution, zero or one occurrence per sequence; background model, 0-order model of sequences; and motif width, 10–16. The probability matrix corresponding to the conserved motif with the most significant (lowest) *E*-value that included the HutC binding palindromic sequence was chosen for further analysis using the FIMO program in the MEME suite (69). The PAO1 genome was then scanned for motif occurrences with a *P*-value threshold of less than 0.0001.

Statistical analyses were conducted using the built-in tools of GraphPad Prism v10. For phenotypic comparisons between wild-type and its isogenic mutant, multiple unpaired *t* tests were performed using the Holm-Šídák method with a significance threshold of 0.05. Alternatively, two-way analysis of variance was used to examine the effects of additional treatment factors, such as time and nutrient substrate.

## Data Availability

The RNA-seq data have been deposited in NCBI’s Gene Expression Omnibus with accession number GSE312996.

## SUPPLEMENTAL MATERIAL

Supplemental material mbio.03886-25-s0001.pdf Fig. S1-S14; Table S1-S3.

ASM does not own the copyrights to Supplemental Material that may be linked to, or accessed through, an article. The authors have granted ASM a non-exclusive, world-wide license to publish the Supplemental Material files. Please contact the corresponding author directly for reuse.
