# Supplementary material for: Global regulation and virulence mediated by the histidine-responsive local transcription factor HutC in Pseudomonas aeruginosa
Source: mBio. 2026 Feb 5;17(3):e03886-25. doi: 10.1128/mbio.03886-25 (PMC12977541; doi:10.1128/mbio.03886-25)
Supplement: Supplemental material — Fig. S1-S14; Table S1-S3. [file mbio.03886-25-s0001.pdf]

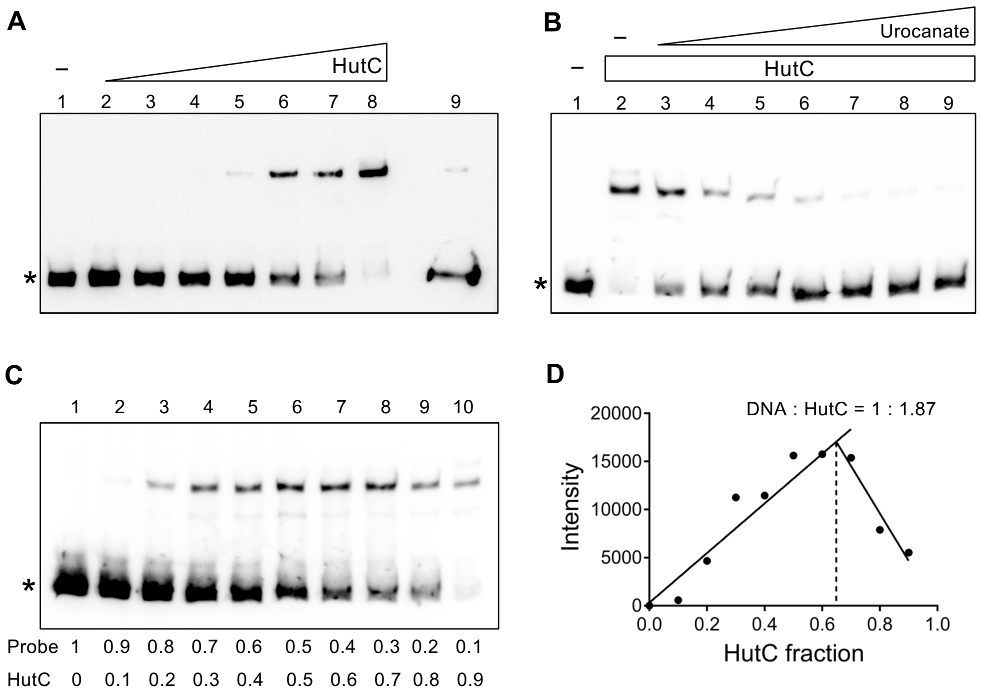

Figure S1. Molecular interactions between His$_6$-tagged HutC$_{PAO1}$ and P$_{hutF}$ promoter DNA from *P. aeruginosa* PAO1.

(A) EMSA with biotin-labeled probe PhutFC-176. HutC$_{PAO1}$ was added at increasing concentrations of 0, 200, 400, 600, 800, 1000, 1400, and 2000 nM in lanes 1 to 8, respectively. Lane 9 contains 20-fold excess unlabeled probe as specific competitor.

(B) EMSA with HutC (1400 nM) and urocanate added at increasing concentration of 0, 0.05, 0.1, 0.2, 0.3, 0.4, 0.6, and 1.0 mM in lanes 2 to 9, respectively.

(C) EMSA using varying ratios of HutC protein and the PhutFC-176 probe DNA, as indicated beneath the gel image. The total molar concentration was held constant at 400 nM.

(D) Job plot of DNA-to-protein ratios for the dominant shifted band in EMSA (C). The intersection of the lines fitted by least-squares to the rising and falling subsets of data indicates the binding stoichiometry of protein-DNA complex.

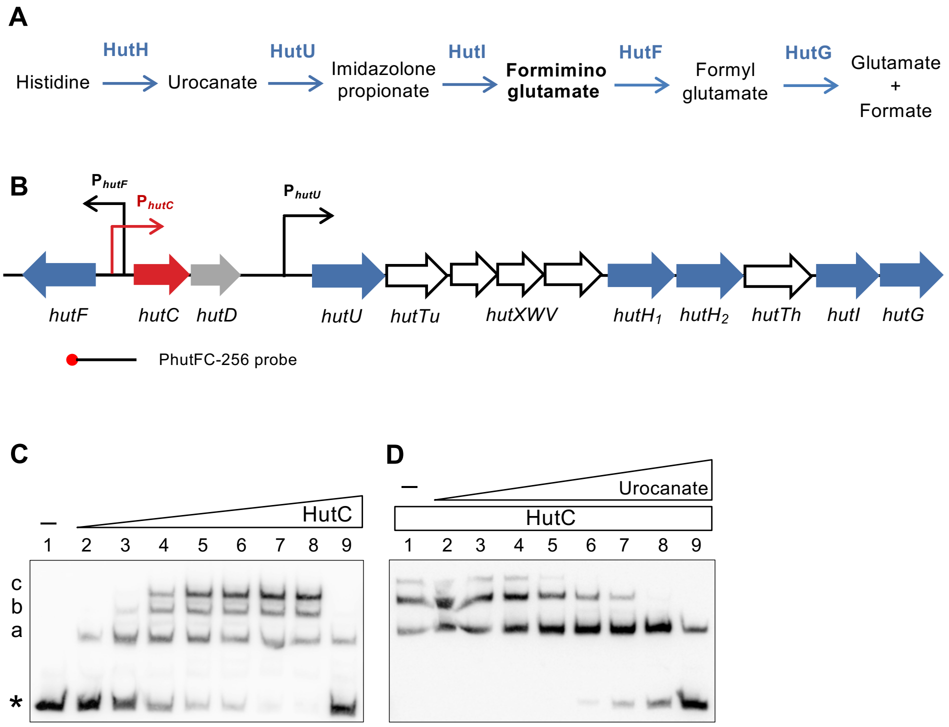

E

Protein-DNA complex	Migration distance (cm)	Molecular weight of HutC proteins (kDa)	Number of HutC proteins
c	4.4	153.13	5.53
b	5.00	113.75	4.11
a	6.00	65.63	2.37
Free probe	8.25		

Figure S2. Molecular interactions between HutC and P$_{hutF}$ promoter in *P. fluorescens* SBW25.

(A) The histidine pathway of *P. fluorescens* SBW25 lacking the HutE-mediated branch for FIGLU degradation.

(B) Genetic map of the *hut* locus in *P. fluorescens* SBW25. The histidine utilization genes are organized in three transcriptional units. The PhutFC-256 probe DNA was labelled with biotin at the 5' end, represented with a red circle.

(C) EMSA with the PhutFC-256 probe DNA. His$_6$-tagged HutC$_{SBW25}$ was added at increasing concentrations of 0, 35, 70, 140, 210, 280, 350, 455, and 455 nM in lanes 1 to 9, respectively. A 200-fold molar excess of unlabeled PhutFC-256 probe DNA was added as a specific competitor in lane 9.

(D) EMSA showing the effects of urocanate on the interactions between HutC$_{SBW25}$ (350 nM) and PhutFC-256 probe DNA (20 nM). Urocanate was added at the final concentration of 0, 0.05, 0.5, 5, 50, 125, 250, 750 μM, and 2.5 mM in lanes 1 to 9, respectively.

(E) Stoichiometric analysis using the Hilmar Bading's method for the three dominant shifted bands.

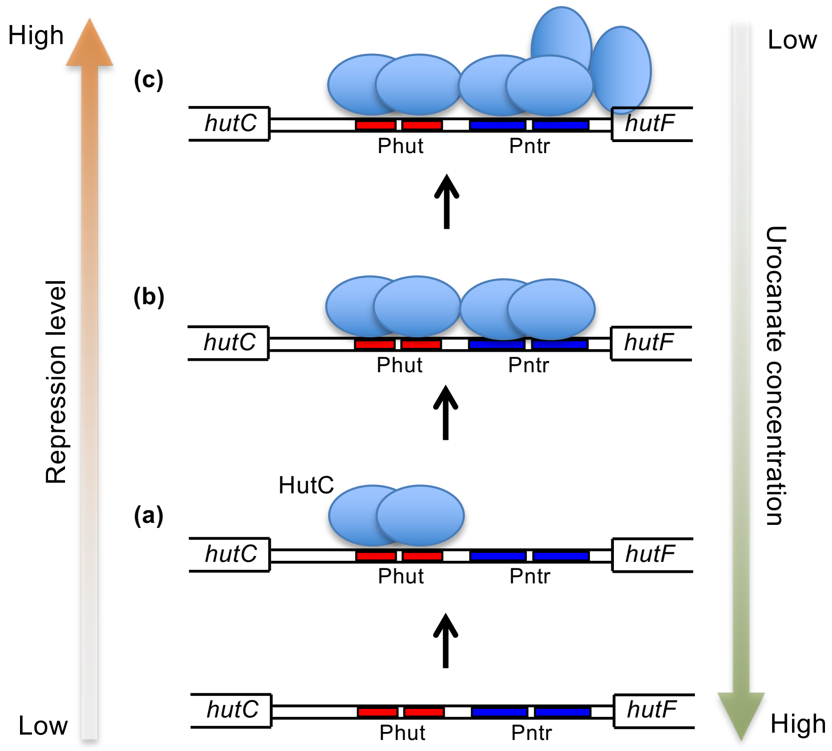

Figure S3. Schematic model of HutC functioning at the promoter region of the *hutF* gene in *P. fluorescens* SBW25.

HutC dissociates from the P_{hutF} DNA through specific interactions with urocanate. As urocanate levels decrease, HutC first forms a relatively stable dimer, with each monomer binding to one half-site of the strong Phut operator (a). Subsequently, HutC binds as a dimer to the adjacent Pntr site, resulting in the presence of two homodimers in the intergenic region between *hutF* and *hutC* genes (b). A further decrease in urocanate concentration leads to the formation of a HutC hexamer, resulting in maximal repression (c).

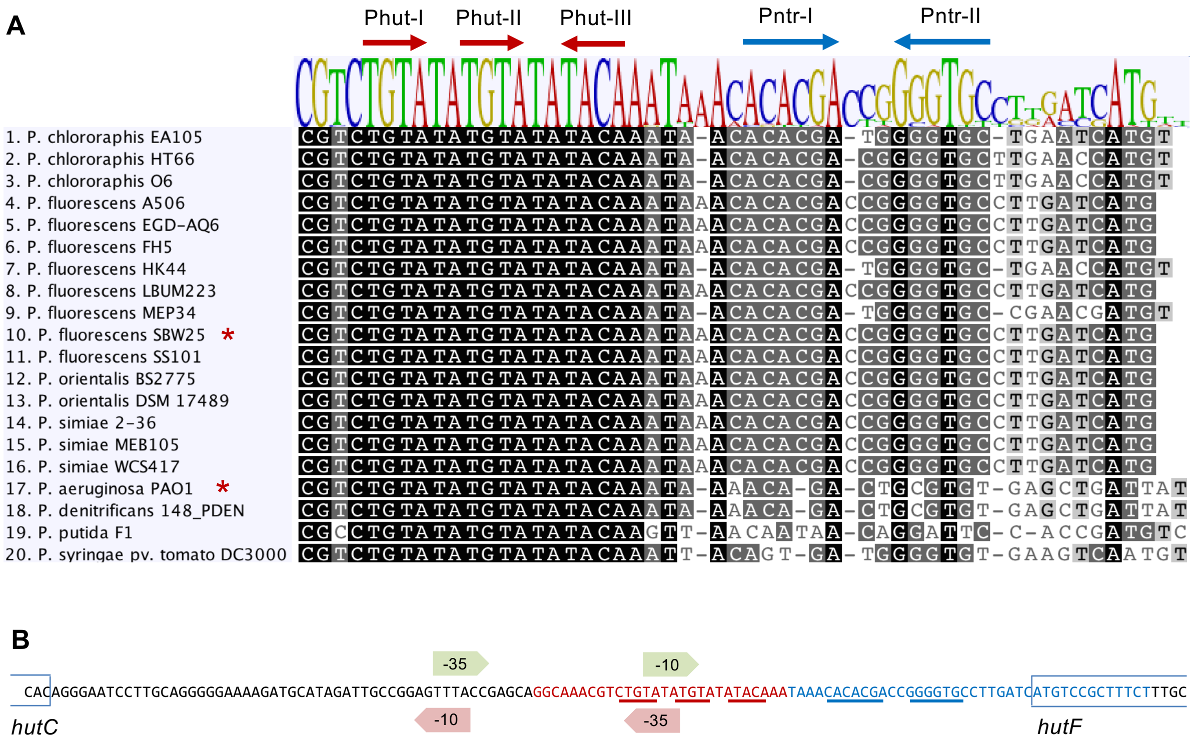

Figure S4. Distribution of the noncanonical HutC-binding site in *Pseudomonas.*

(A) Alignment of *hutF* promoter sequences performed in Geneious 9.0.5 (Biomatters, Auckland, New Zealand). The Phut and Pntr half sites are indicated by red and blue arrow, respectively.

(B) DNA sequences of intergenic region between *hutF* and *hutC* genes in *P. fluorescens* SBW25. The strong and weak HutC binding regions are shown in red and blue font, respectively. The -35 and -10 elements for the transcription of *hutF* and *hutC* are denoted by arrow bars.

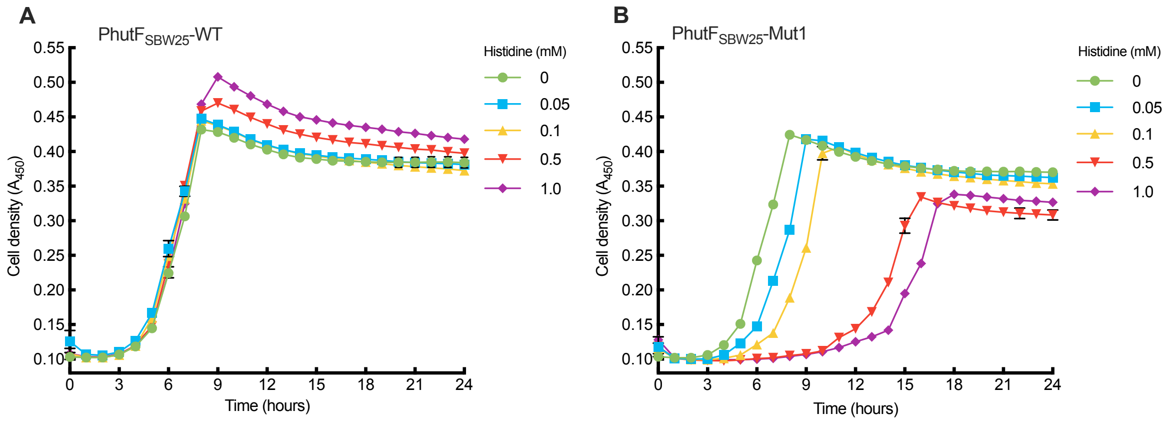

Figure S5. Histidine concentration-dependent growth inhibition in *P. fluorescens* SBW25 lacking the noncanonical Pntr site in the *hutF* promoter.

Bacteria were grown in M9 salt medium supplemented with 5 mM glutamate and varying concentrations of histidine as the sole carbon and nitrogen source.

(A) SBW25 with the wild-type *hutF* promoter (PhutF$_{SBW25}$-WT).

(B) SBW25 carrying P_{hutF} promoter variant (PhutF$_{SBW25}$-Mut1, strain MU59-86), in which the Pntr site was replaced with the corresponding sequence from the *hutF* promoter of *P. aeruginosa* PAO1.

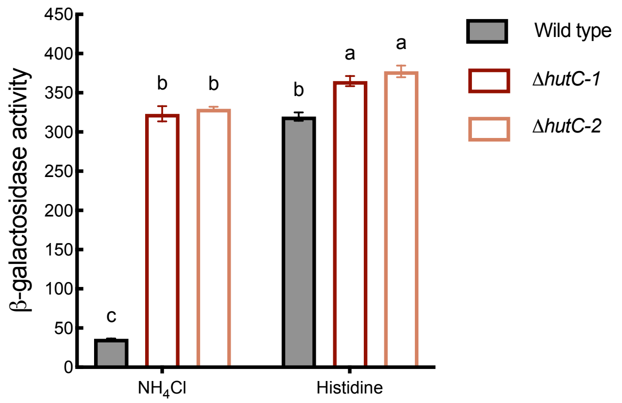

Figure S6. Functional verification of *hutC* deletion mutants.

β-galactosidase assays were performed with wild-type PAO1 and two independently constructed Δ*hutC* mutants, each carrying a P_{hutU}-*lacZ* fusion integrated at the *att*Tn7 site. β-galactosidase activity (μM 4MU/A_{600}/min) was measured for bacteria grown in M9 salt medium supplemented with succinate (20 mM) as the carbon source and either ammonium chloride or histidine (10 mM) as the nitrogen source. The results showed that P_{hutU} activity was inducible by histidine in the wild-type strain, whereas P_{hutU} expression was constitutive in the Δ*hutC* mutants. As expected, P_{hutU} promoter activity was significantly higher in the Δ*hutC* mutants compared to the wild type in the presence of histidine.

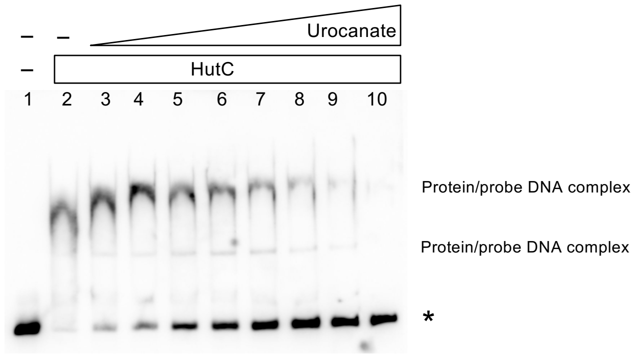

Figure S7. Effects of urocanate on HutC binding to the P_{arr} promoter in *P. aeruginosa* PAO1.

EMSA performed using His_6-tagged $HutC_{PAO1}$ and a biotin-labelled DNA probe “Parr-246” corresponding to the *arr* promoter region. In lanes 2 to 10, $HutC_{PAO1}$ was included at a constant concentration of 3.2 μM, while urocanate was added at increasing concentration of 0, 25, 50, 75, 100, 200, 400, 600, and 1000 μM. The asterisk indicate the position of the free probe DNA.

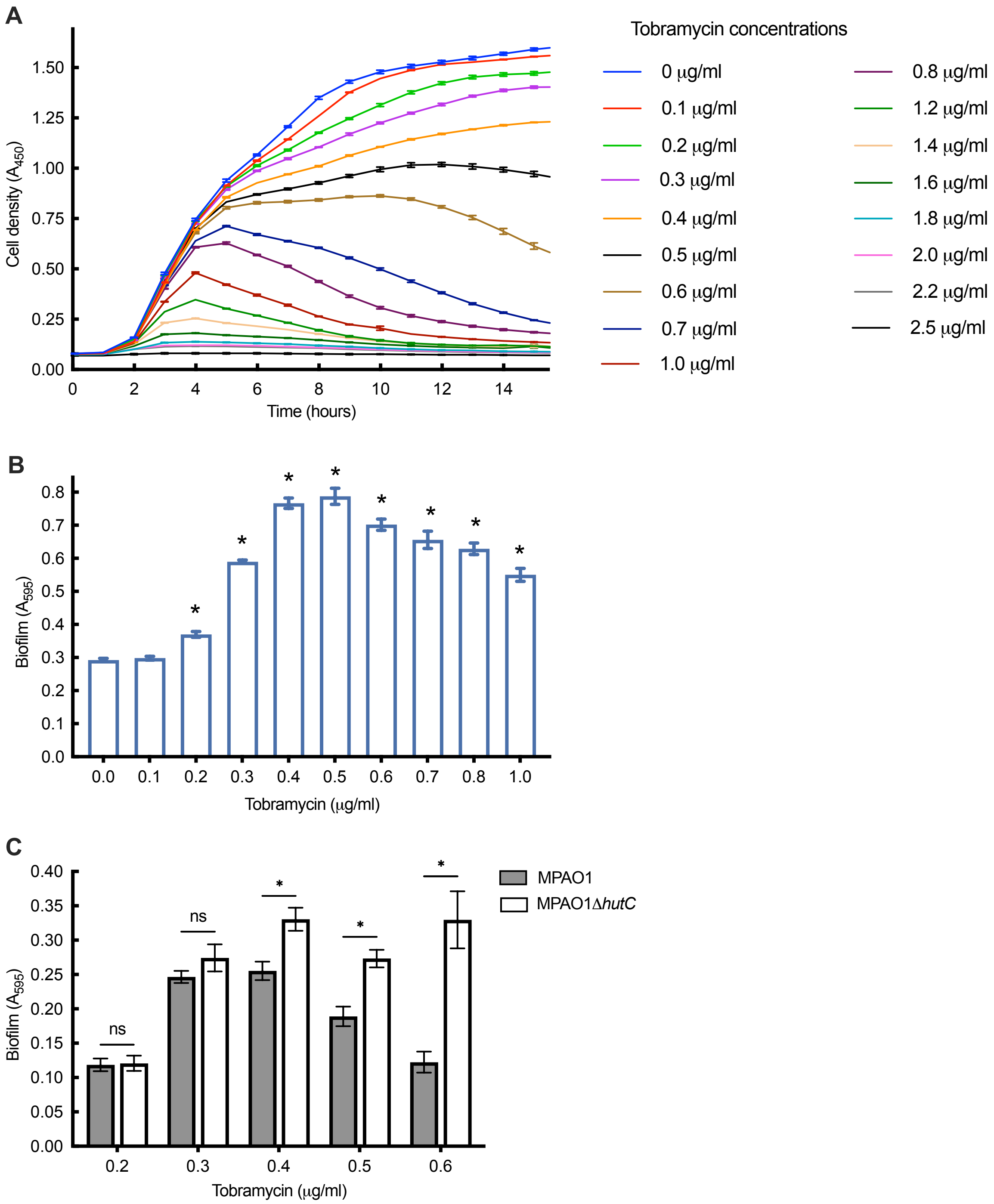

Figure S8. Tobramycin-induced biofilm formation in *P. aeruginosa* MPAO1.

(A) Growth curves of wild-type MPAO1 in LB broth supplemented with varying concentrations of tobramycin. Data are means and standard errors of four replicate cultures.

(B) MPAO1 biofilm expressed as absorbance at 595 nm (A_{595}). Bacteria were grown in static LB broth in the presence of tobramycin, and biofilm levels were quantified 8 hrs after inoculation. Data are means and standard errors of 8 replicate cultures. Asterisk indicates a significant difference compared with non-antibiotic control ($P < 0.0001$), Student's t-test).

(C) Deletion of *hutC* caused increased tobramycin-induced biofilm formation. The same data shown in Figure 5D is re-plotted by extracting the amount of biofilm (A_{590}) formed in the absence of tobramycin, thereby representing the tobramycin-induced biofilm levels. Asterisk and "ns" denote significant and non-significant difference, respectively ($P < 0.05$).

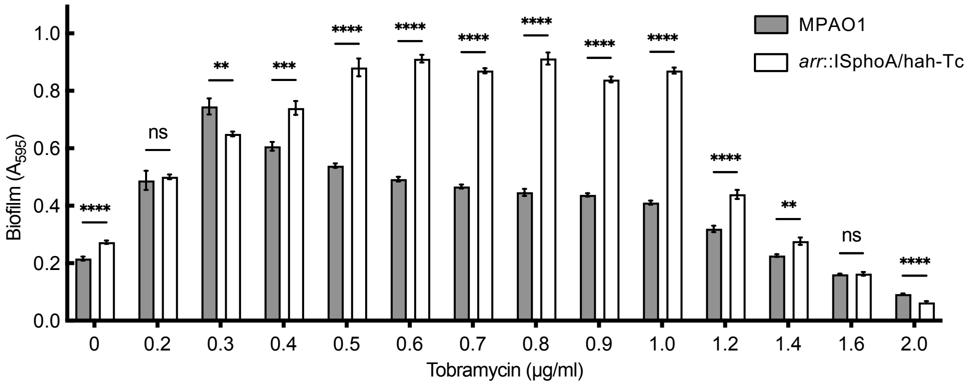

Figure S9. Tobramycin-induced biofilm formation by wild-type MPAO1 and one additional *arr* inactivation mutant MU61-32 (*arr*::ISphoA/hah-Tc).

Biofilm expressed as absorbance at 595 nm (A_{595}) was quantified by growing bacteria in static LB broth 8 hrs after inoculation. Data are means and standard errors of 8 replicate cultures. Asterisks denote significant difference at $P < 0.05$ (*), 0.01 (**), 0.001 (***), and 0.0001 (****). ns, not significant at $P < 0.05$.

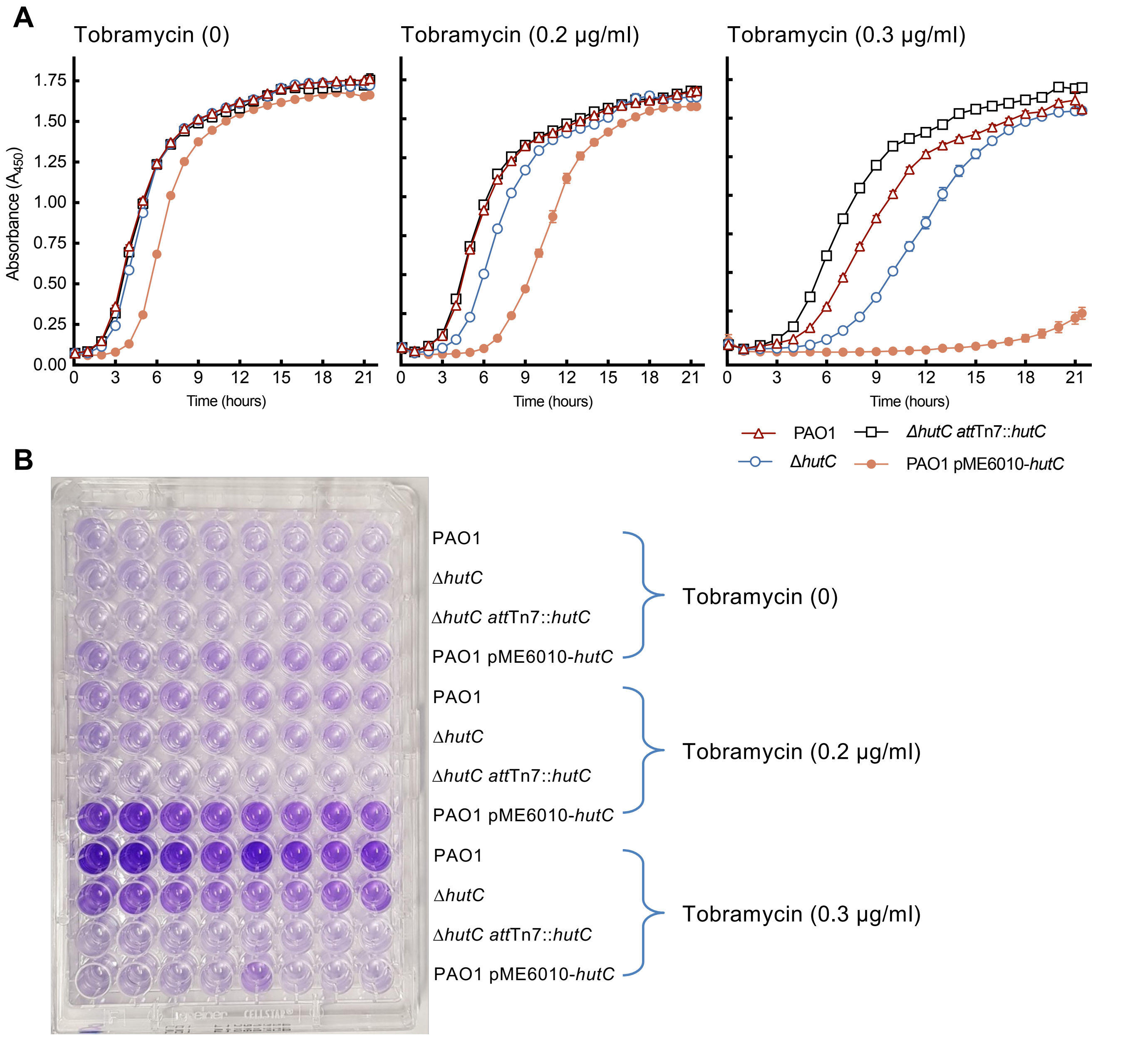

Figure S10. Tobramycin-induced biofilm formation by wild-type PAO1 and its derived *hutC* mutants.

(A) Planktonic growth measured in shaken 96-well microtiter plate. Data re means and standard errors of eight replicate cultures. Absorbance data are plotted by the tobramycin treatment with the final concentration of 0, 0.2, and 0.3 µg/ml.

(B) Microtiter plate with biofilm being stained and solubilized for the measurement of absorbance (A_{595}). The corresponding absorbance data are shown in Figure 5E.

The four *P. aeruginosa* strains are wild-type PAO1, Δ*hutC*, MU42-29 for *hutC* complementation (Δ*hutC att*Tn7::*hutC*), and MU50-19 for *hutC* over-expression (PAO1 pME6010-*hutC*). Biofilm formation was quantified 8 hours after inoculation in Mueller-Hinton Broth (MHB) medium.

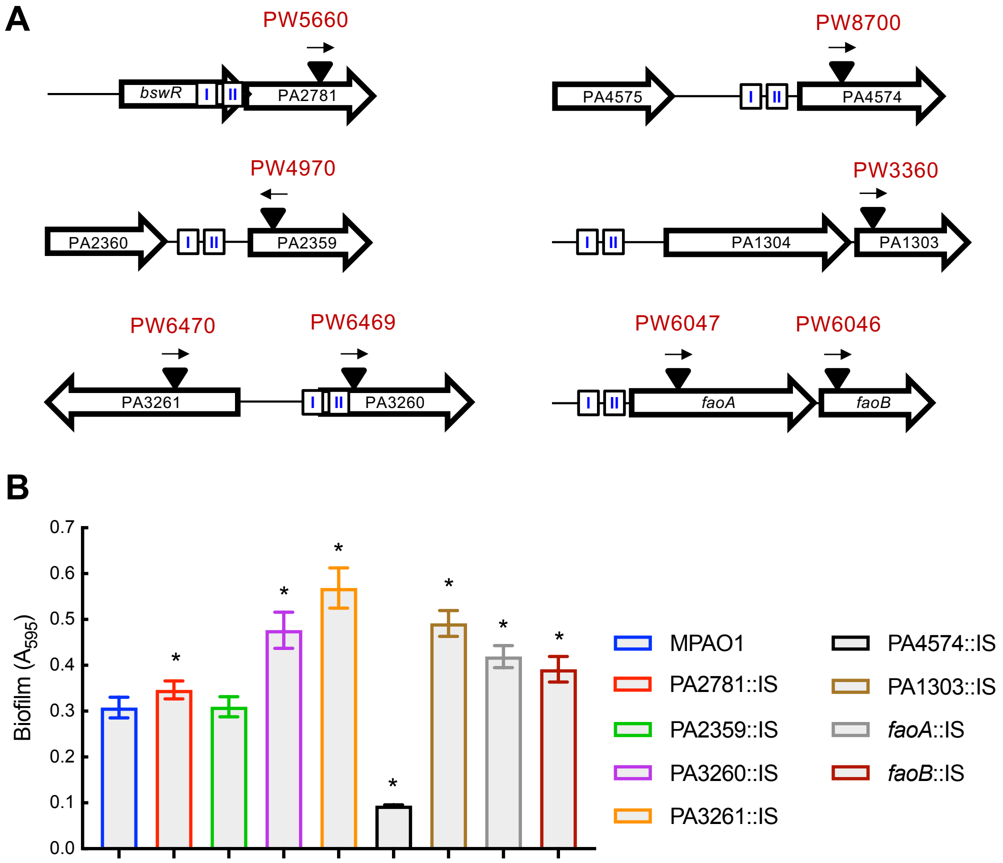

Figure S11. Assessment of biofilm formation in MPAO1 mutants with IS insertions in HutC-targeted genes.

(A) Schematic representation of the genetic loci targeted by HutC. The two putative HutC-binding half sites (Phut-I and Phut-II) are marked in blue. Black inverted triangles indicate locations of transposon insertions, with arrows above the triangles denoting orientation of the inserted transposon. The related strain names or PW numbers in the transposon library at the University of Washington are shown in red font.

(B) Comparison of biofilm formation by MPAO1 and its derived mutants carrying insertions of either ISphoA/hah-Tc or ISlacZ/hah-Tc. Bacteria were grown statically in LB broth medium for 16 hours in 96-well microtiter plates. Data are means and standard deviations of eight replicate cultures. Asterisks indicate statistically significant difference in biofilm formation relative to the wild-type strain, as determined by Dunnett's multiple comparison test ($P < 0.05$).

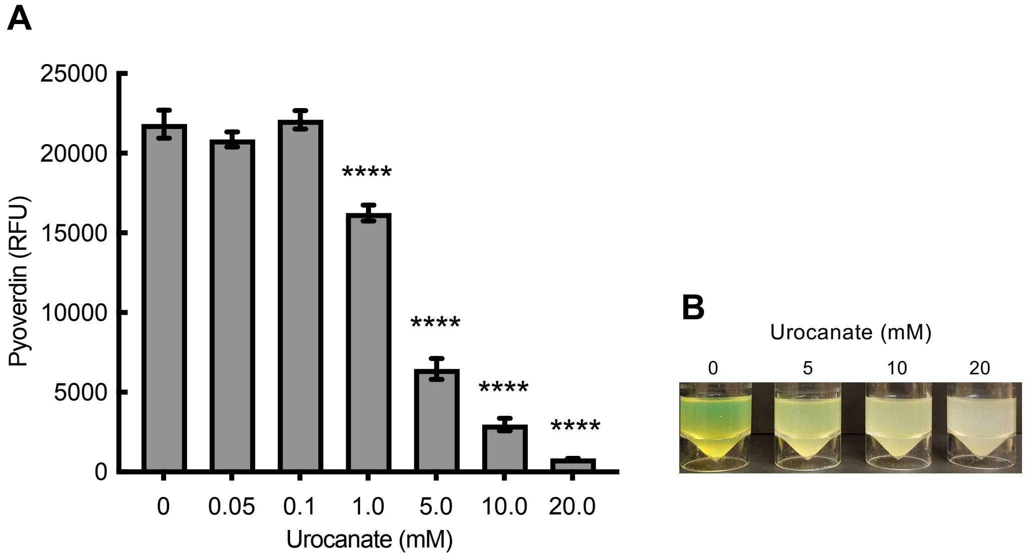

Figure S12. Concentration-dependent inhibition of pyoverdine production by urocanate.

(A) Wild-type MPAO1 was grown in "MSM + succinate + NH_4Cl" supplemented with varying concentrations of urocanate. Pyoverdine levels were estimated by measuring fluorescence at 460 nm with an excitation wavelength of 365 nM at 16 hours after inoculation. Data are means and standard errors of four independent cultures. Asterisks indicate statistical significance compared with the non-urocanate control ($P < 0.0001$), as determined by Dunnett's multiple comparison test.

(B) Visual comparison of fluorescent pigment for wild-type MPAO1 grown in "MSM + succinate + NH_4Cl" with urocanate added at different concentrations.

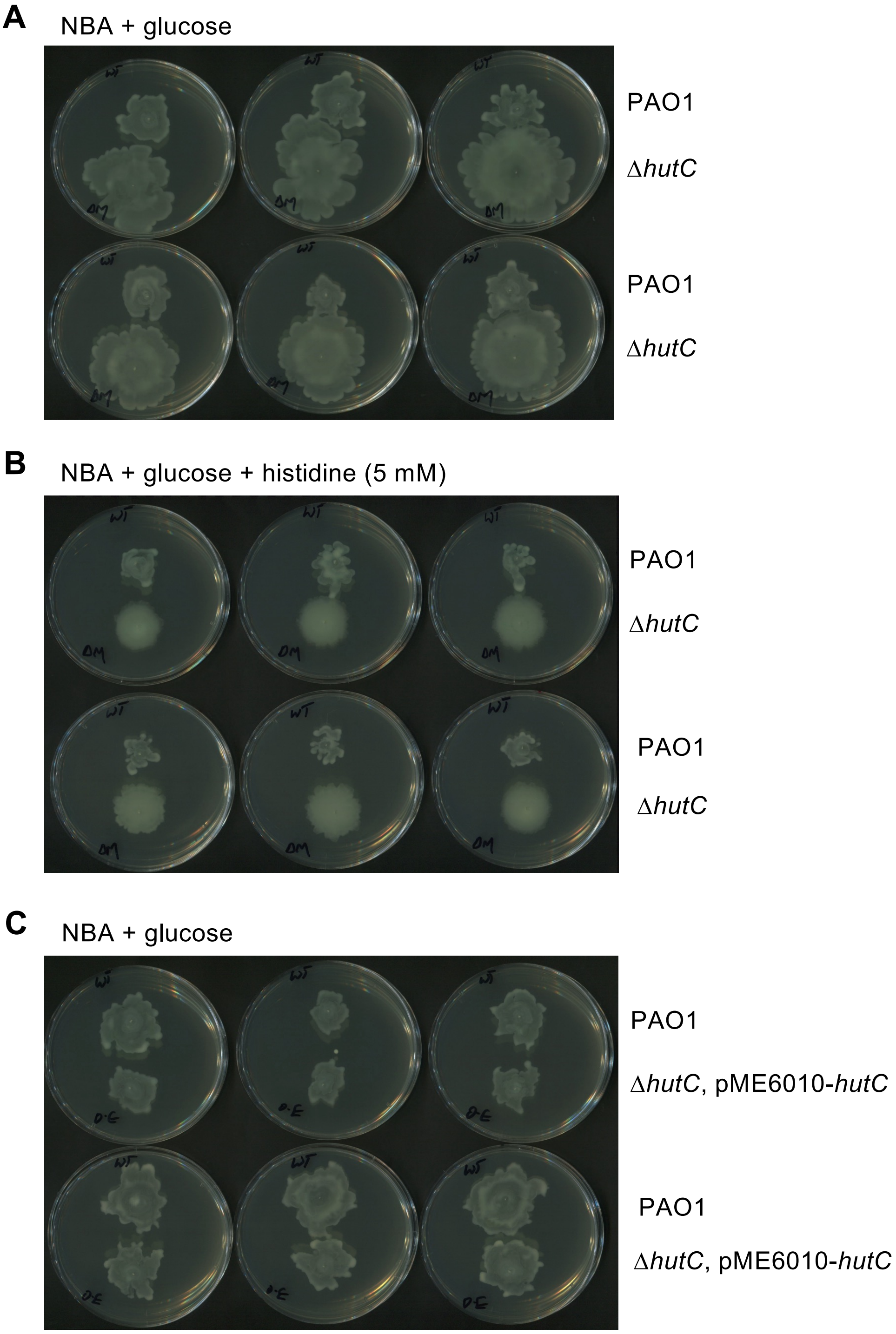

Figure S13. Swarming of PAO1 and its derived mutants on 0.5% nutrient broth agar (NBA) supplemented with glucose.

Photographs were taken 24 hours after inoculation at 30°C. Positions of the *P. aeruginosa* strains are indicated at the right-hand side of the photograph.

(A) Similar to the MPO1-derived Δ*hutC* mutant, deletion of *hutC* in strain PAO1 also resulted in a hyper-swarming phenotype.

(B) Addition of histidine altered the patterns of swarming for the Δ*hutC* mutant.

(C) Over-expression of *hutC* (strain MU50-19) abolished the super-swarming phenotype of the Δ*hutC* mutant.

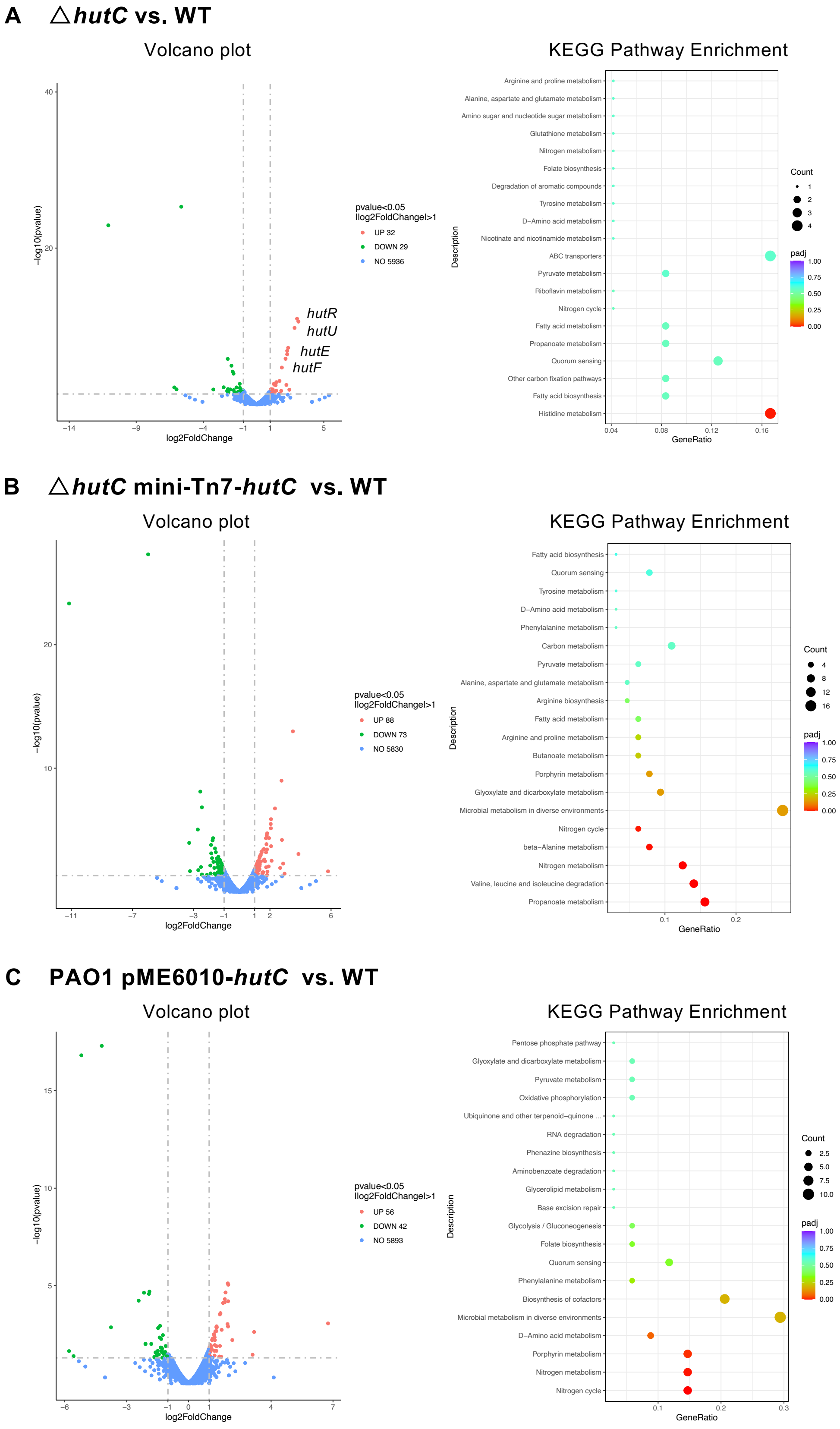

Figure S14. Volcano plot and KEGG pathways enriched in *P. aeruginosa* mutants for cells grown on histidine as the sole source of carbon and nitrogen.

In the volcano plots showing the differentially expressed genes (DEGs), red dots represent genes upregulated in the mutants, whereas green dots indicate genes downregulated relative to wild-type PAO1 (WT). In the KEGG pathway analysis, bubble size reflects the number of DEGs associated with each pathway, while the colour indicates the degree of pathway enrichment. **(A)** Strain MU57-46 (Δ*hutC*) versus wild-type PAO1 (WT). **(B)** Strain MU42-29 (Δ*hutC,* mini-Tn7T-*hutC*) versus wild type. **(C)** Strain MU50-19 (PAO1, pME6010-*hutC*) with constitutive *hutC* over-expression versus wild type.

Supplementary Data

Table S1. Dissociation constant (K_d) between HutC$_{PAO1}$ and its target promoters in *P. aeruginosa* PAO1

Promoter	DNA probe	Binding affinity (K_d)	HutC concentrations used in EMSAs
hutU	PhutU-191	251 ± 13.09 nM	0, 70, 140, 200, 300, 400, 600, 800, and 1200 nM from lanes 1 to 9 (Fig. 1D)
hutF and *hutC*	PhutFC-176	1.34 ± 0.11 µM	0, 200, 400, 800, 1000, 1400, and 2000 nM from lanes 1 to 8 (Fig. S1A)
faoA	PfaoAB-207	6.57 ± 0.14 µM	0, 4.0, 5.0, 6.0, 8.0, 10.0, 12.0, and 15.0 µM from lanes 1 to 8 (Fig. 4)
cupA1 and *cgrA*	PcupA1-cgrA-228	4.13 ± 0.02 µM	0, 1.0, 2.0, 3.0, 4.0, 6.0, 7.5, and 12.0 µM from lanes 1 to 8 (Fig. 4)
nadB and *algU*	PnadB-algU-184	5.84 ± 0.03 µM	0, 1.0, 2.0, 3.0, 4.0, 6.0, 7.5, and 10.0 µM from lanes 1 to 8 (Fig. 4)
PA2781	P$_{PA2781}$-205	4.45 ± 0.18 µM	0, 0.5, 1.0, 1.5, 2.0, 3.0, 4.0, and 5.0 µM from lanes 1 to 8 (Fig. 4)
PA1292	P$_{PA1292}$-268		0, 0.5, 1.0, 2.0, 2.5, 3.0, 3.5, and 4.0 µM from lanes 1 to 8 (Fig. 4)
arr	Parr-246	5.68 ± 0.62 µM	0, 0.5, 1.0, 1.5, 2.0, 3.0, 4.0, and 5.0 µM from lanes 1 to 8 (Fig. 4)

Table S2. Oligonucleotides used in this work

Primer	Sequence (5' - 3')[a]	Application
P. aeruginosa		
hutC-del-F1	cagatctATGCGCAGGGAAAGTTCGGC	Δ*hutC*, mutants MU61-68 and MU57-46 construction
hutC-del-R1	ccagacggtgTACCTCGCCGAGTTGCGAG	
hutC-del-F2	cggcgaggtaCACCGTCTGGAAGGACGTT	
hutC-del-R2	gagatctTACCCTTTCGGTGTAGACGC	
arr-del-1	cagatCTGCTCGTCCTGGTCTCCTT	Δ*arr*, mutant MU61-71 construction
arr-del-2	ctcgccgctaCATCCTGGCCTGCTGGTACA	
arr-del-3	ggccaggatgTAGCGGCGAGCTAGAGGGGCAGAT	
arr-del-4	gagatCTCCTACCTCGAGCACCACCT	
PAO1-HutCF	ggaattccataTGACGTCCTCTTCCTCCGA	HutC$_{PAO1}$ protein expression
PAO1-HutCR	ctggatccTCATGAGCTGAAACGTCCTTCC	
PAO1-hutCF	aactagtGGACATTTCGCGCCCAGCCT	Amplifying DNA probe "PhutFC-176"
PhutFC-bio1[b]	GGAGGAAGAGGACGTCAC	
PhutU-bio1	CGGGAATGTGGAGATATCG	Amplifying DNA probe "PhutU-191"
MLG093-PR	cacaagcttCGGGGTGGTCACGGCAGGCTCCTG	
PPntr-1	gagatctACGATCAGCGAGTGGAACAC	Strain MU63-95 construction for incorporation of a Pntr site in the *hutF* promoter of PAO1
PPntr-2	tcaaggcaccccggtcgTGTTTTATTTGTATATACATATAC	
PPntr-3	acacgaccggggtgcctTGATTATGTCCGCAATTTTCG	
PPntr-4	gagatctGAAGCCTGCGTGACTGTAAAG	
KSJ-111-F-bio[b]	TGAAAGAGACTGTCCCGCCG	

KSJ-012-R	GGCTTCTGGGGTAAGATTCG	Amplifying DNA probe “Parr-246” for the *arr* promoter
KSJ-009-F	CTCATCCTCAAGGTGTCCAG	Amplifying DNA probe “P_{PA2781}-205” targeting the intergenic region between *bswR* and PA2781
KSJ-010-R-bio[b]	TACTGCTCCGGATGCGTGTC	
foaA-1	GCGCAGGCCTCGACAATAGA	Amplifying DNA probe “PfaoAB-207” for the *faoAB* promoter
foaA-2-bio[b]	TCAACTCGACGATGCCGC	
CupA-1-bio[b]	CGTTCGAGTACCCGCCATTC	Amplifying DNA probe “PcupA1-cgrA-228” for EMSA of intergenic region between *cupA* and *cgrA* genes
CupA-2	CAGGGAAGTGCATACTCCAAC	
AlgU-F-bio	GCTGCCGATGACCAGTACAT	Amplifying DNA probe “PnadB-algU-184” for EMSA of intergenic region between *algU* and *nadB* genes
AlgU-R	GTTCCAGCACTACATCGCC	
p3MST-F	ACGACATCGCGGACTCCTTG	Amplifying DNA probe “P_{PA1292}-268” for EMSA of PA1292 promoter
p3MST-R-bio[b]	CCAAGCCAATCGGACAAGC	
PAO1-hutCF	aactagtGGACATTTCGCGCCCAGCCT	*hutC* gene complementation at the *att*Tn7 site
PAO1-hutCR	aaaGCTTGAAGCCGTCCAGGCCGTCG	
KSJ007-F	ggagatctCTCACTTCATCGACGCCTCG	Amplifying *hutC* gene for overexpression in pME6010
KSJ008-R	ggaagcttGGATGCGGAGTTCGCTCATG	
PAO1-glmS	CAACCTGGCCAAGTCGGTCACC	Verifying chromosomal integration at the *att*Tn7 site
Tn7R109	CAGCATAACTGGACTGATTTCAG	
P. fluorescens		
hutF-new1	gagatCTCGGCGACGAAGGTGCCCA	Δ*hutF*, mutant MU35-86 construction
hutF-new2	cagcatgcggatccgttgacggaCAGCCCATCGGCGCTGACTT	
hutF-new3	tccgtcaacggatccgcatgctgTGGGCTAACCGGCAACCCAT	
hutF-new4	gagatctAGCCAAGGGCACGGCTACG	
SPntr-1	gagatctGGTGAAATCCTGCTGCAGGT	Strain MU59-86 construction for the elimination of the Pntr site in the *hutF* promoter
SPntr-2	tcagctcacacgcagtcTGTGTTTATTTGTATATACA	
SPntr-3	acagactgcgtgtgagcTGATCATGTCCGCTTTCTTTG	
hutC-1	agatctGAGTGGAAGCACAGGCCCA	
Bio-F-new2[b]	CAGCCCATCGGCGCTGACTT	Amplifying probe DNA “PhutFC-256”
hutF-F	aactagtACAAGGGCGCCGGACTTTCG	
hutF1	gaagatcTGATCTGACGCGACAGTTC	Sit-directed mutagenesis of the Pntr site of the *hutF* promoter to produce the “PhutFSBW25-Mut2” variant
hutF-M1	aacatgagcccggcagaaCTTGATCATGTCCGCTTTCTT	
hutF-M2	aagttctgccgggctcatGTTTATTTGTATATACATATAC	
hutC5	cgggatcCTCTTGGGCTCGGCGACGA	
hutC-2	agcatgcgatcgttgacggaCTCTTGGGCTCGGCGACGA	*hutF* complementation
hutF-2	cgggatccGGTTAGCCCAGCAAATC	
SBW25-glmS	CACCAAAGCTTTCACCACCCAA	Verifying chromosomal integration at the *att*Tn7 site
Tn7R109	CAGCATAACTGGACTGATTTCAG	

[a]Artificial sequences integrated into the primers are shown in lowercase with restriction sites underlined.

[b]Primers are biotin-labelled at the 5’-end.

Table S3. List of putative HutC-binding sites in the genome of *P. aeruginosa* PAO1

Motifs[a]	Motif location		*P* value	Locus tag and gene/protein information
	Start	End		
TACTTGTATGTACAAG	5744858	5744873	3.89E-10	PA5100: *hutU*
AGCTTCTATATACAAG	1757703	1757718	1.26E-06	PA1613: hypothetical protein;<br>PA1614: glycerol-3-phosphate dehydrogenase
GGCTGGTAGGTACAAG	5629913	5629928	5.42E-06	PA5011: *waaC*, heptosyltransferase I;<br>PA5010: *waaG*, UDP-glucose:(heptosyl) LPS alpha 1,3-glucosyltransferase
TGCCTGTGTGTCCAAG	4198908	4198923	6.31E-06	PA3747: conserved hypothetical protein;<br>PA3746: *ffh*, signal recognition particle protein
TGCTTGTATTTCAAC	3173249	3173264	9.03E-06	PA2818: *arr*, aminoglycoside response regulator
TACGGGTATATACACG	1396746	1396761	1.04E-05	PA1285: probable transcriptional regulator;<br>PA1286: major facilitator superfamily transporter
TATATGTATATACAAA	5749411	5749426	1.32E-05	PA5106: *hutF*<br>PA5105: *hutC*
TGTTTTTGTATACAAG	537923	537938	1.71E-05	PA0476: permease
TCCTTGTGCGTACAGG	3138168	3138183	1.80E-05	PA2781: hypothetical protein;<br>PA2780: *bsWR*, bacterial swarming regulator
TCCTTGTAAGCACAAA	2549639	2549654	1.94E-05	PA2311: hypothetical proten;<br>PA2310: hypothetical protein
TTGTTGTAGGTGCAAG	2766998	2767013	1.96E-05	PA2462: probable haemagglutinin
TCCTTGTGTGTGCAAC	3930462	3930477	2.04E-05	PA3514: ATP-binding component of ABC transporter
AACTTGAATGTGCAGG	5928562	5928577	2.27E-05	PA5265: hypothetical protein
GACATGAATGTCCAAG	3648054	3648069	2.35E-05	PA3260: probable transcriptional regulator;<br>PA3261: hypothetical protein
TACAGGTATGAACATG	3549787	3549802	2.38E-05	PA3162: *rspA*, 30S ribosomal subunit
TACGGGCATGTACAGG	2084259	2084274	2.49E-05	PA1910: *femA*, ferric mycobactin receptor
GACCTGTGTGTGCAAG	5355312	5355327	2.49E-05	PA4769: probable transcriptional regulator;<br>PA4770: *lldP*, lactate permease
TACGTGGATGTGCATG	4499829	4499844	2.53E-05	PA4020: *mpl*, UDP-N-acetylmuramate: L-alanyl-gamma-D-glutamyl-meso-diaminopimelate ligase
TACTGGTAGGTGCATG	5172764	5172779	2.53E-05	PA4613: *katB,* catalase
CACCGGTATGAACAAG	6136787	6136802	2.58E-05	PA5447: *wbpZ*, lipopolysaccharide biosynthesis
CACCTGGATGTACAGG	5170863	5170878	2.67E-05	PA4613: *katB*, catalase;<br>PA4612: hypothetical protein
TTTTTGTTTTTACAAG	3685174	3685189	2.74E-05	PA3291: *til1*;<br>PA3292: hypothetical protein
CACCTGCCTGTACAAG	657340	657355	2.81E-05	PA0596: aminoglycoside phophotransferase;<br>PA0595: *lptD*, LPS assembly protein
TAGATGTGTGTACAGG	1985456	1985471	2.94E-05	PA1827: probable short chain-dehydrogenase, fatty acid metabolism
TACTTGTAGGTGCAGC	2289653	2289668	3.35E-05	PA2080: *kynU*, kynureninase, Tryptophan metabolism;<br>PA2079: probable amino acid permease
TAATGGCCTGTACAAG	3122777	3122792	3.54E-05	PA2763: hypothetical protein
TACATCGATGTTCAAG	5174644	5174659	3.67E-05	PA4616: C4-dicarboxylate binding protein;<br>PA4617: a hypothetical protein
GACGTGCATGTACAAC	4840089	4840104	3.75E-05	PA4311: glycoside hydrolase;<br>PA4312: hypothetical protein
AACTTGCATGTCCAAC	5182260	5182275	3.90E-05	PA4621: probable oxidoreductase;<br>PA4622: major facilitator superfamily transporter
TACTTGCATGAATTAG	3683857	3683872	4.03E-05	PA3290: *tle1*, hydrolase
TACTGGTATGTTTCAG	259666	259681	4.15E-05	PA0230: *pcaB*, 3-carboxy-cis, cis-muconate cycloisomerase
TACTTGTACGTTCCAT	654607	654622	4.22E-05	PA0595: *ostA*, LPS assembly protein<br>PA0594: *surA*, peptidyl-prolyl cis-trans isomerase
TACATTTATGAAGAAG	1246585	1246600	4.49E-05	
CGCATGAATGAACAAG	1416960	1416975	4.56E-05	PA1305: hypothetical protein;<br>PA1304: probable oligopeptidase

TGCTTGCGAGAACAAG	1103989	1104004	4.73E-05	PA1019: *mucK*, cis,cis-muconate transporter
TGCTTGATTGAACAGG	237162	237177	4.83E-05	PA0208: *mdcA*, malonate decarboxylase alpha subunit
TGCTGGGATGAACCAG	4766004	4766019	4.93E-05	PA4261: *rplW*, 50S ribosomal protein L23;<br>PA4260: *rpiB*, 50S ribosomal protein L2
TACTTCTATGTGAACG	5550895	5550910	4.93E-05	PA4946: *mutL*, DNA mismatch repair protein
TGCCGGGATGAACAAG	403990	404005	4.93E-05	PA0360: hypothetical protein
CGCTGGAATGTACAGG	4961313	4961328	4.93E-05	PA4427: *sspB*, stringent starvation protein C
TGCTTGTGGGTCCAGG	6168711	6168726	5.15E-05	PA5476: *citA*, citrate transporter
TACTTCTATCCACAGG	3561017	3561032	5.23E-05	PA3170: N-ethylammeline chlorohydrolase;<br>PA3171: *ubiG*, 3-demethylubiquinone-9 3-methyltransferase
AGCTCGGATGTACTAG	4101948	4101963	5.42E-05	PA3660: sodium/hydrogen antiporter;<br>PA3661: hypothetical protein
TGCCTGCAGGTACAGG	753190	753205	5.51E-05	PA0690: *pdtA*, phosphate depletion regulated TPS partner A
TGCCTGGCTGGACAAG	3058190	3058205	5.51E-05	PA2704: probable transcriptional regulator
GGCCTGTACGTACAGG	4371161	4371176	5.51E-05	PA3901: *fecA*, Fe(III) dicitrate transport protein
CGCCTGCAAGTACAAG	4274816	4274831	5.63E-05	PA3818: *suhB*, extragenic suprerssor protein;<br>PA3817: probable methyltransferase
TGCTCGCATGATCAAG	3543504	3543519	5.68E-05	PA3158: *wbpB*, UDP-2-acetamido-2-deoxy-d-glucuronic acid 3-dehydrogenase;<br>PA3157: probable acetyltransferase
TGCCGGTCTGTACCAG	4050611	4050626	5.70E-05	PA3615: hypothetical protein
GGCTTGCATGTCCATG	1429003	1429018	5.74E-05	PA1317: *cyoA*, cytochrome o ubiquinol oxidase subunit II;<br>PA1318: *cyoB*, cytochrome o ubiquinol oxidase subunit I
CGCCTGCATGTCCAAG	4123169	4123184	5.79E-05	PA3680: rRNA small subunit methyltransferase J
CGCCTGTATGTCCACG	3802221	3802236	5.79E-05	PA3396: *nosL*, putative nitrous oxide reductase protein
TATTTATATGTGAAAG	4820039	4820054	5.91E-05	PA4295: *fppA*, Fip prepilin peptidase A;<br>PA4296: *pprB* -two-component response regulator
AGCTGGTATCGACAAG	3541132	3541147	6.00E-05	PA3157: probable acetyltransferase;<br>PA3156: *wbpD*, UDP-2-acetamido-3-amino-2,3-dideoxy-d-glucuronic acid N-acetyltransferase
TGCATGTCTGGACAAA	1404013	1404028	6.09E-05	PA1292: probable 3-mercaptopyruvate sulfurtransferase
GGAATGCATGTACAAG	4095558	4095573	6.09E-05	PA3657: *map*, methionine aminopeptidase;<br>PA3658: *glnD*, protein-PII uridylyltransferase;<br>PA3656: *rpsB*, 30S ribosomal protein S2
TGAATGTATGGTCAAG	1483097	1483112	6.16E-05	PA1369: hypothetical protein;<br>PA1368: hypothetical protein
GGCTTGCATGAAGAAG	517382	517397	6.25E-05	PA0458: major facilitator superfamily transporter;<br>PA0459: probable ClpA/B protease ATP binding subunit
AGCTTGTAGGTGCAAT	3343461	3343476	6.33E-05	PA2985: hypothetical protein;<br>PA2984: hypothetical protein
TGCGGGTATGCAGAAG	4625069	4625084	6.41E-05	PA4135: probable transcriptional regulator
TGGTGGGATGTACATG	3325385	3325400	6.51E-05	PA2966: *acpP*, acylcarrier protein;<br>PA2967: *fabG*, 3-oxoacyl-[acyl-carrier-protein] reductase
TGCTTGCGGCTACAAG	4756156	4756171	6.51E-05	PA4239: *rpsD*, 30S ribosomal protein S4;<br>PA4240: *rpsK*, 30S ribosomal protein S11
TGTGTGTATGTGCAGG	5122395	5122410	6.51E-05	PA4574: membrane protein
TGCCTGCATGGAAAAG	995110	995125	6.61E-05	PA0911: *alpE*
TGCCTGAATCCACAAG	174721	174736	6.73E-05	PA0153: *pcaH*, protocatechuate 3,4-dioxygenase, beta subunit
TGATTGCCTGTTCAAG	3406738	3406753	7.02E-05	PA3044: *rocsS2*, two component sensor
TGCTTTCCTGTACAGG	1515487	1515502	7.14E-05	PA1393: *cysC*, adenosine 5'-phosphosulfate (APS) kinase
TGCTCCTTTGGACAAG	2824541	2824556	7.14E-05	PA2505: *opdT*, tyrosine porin OpdT
TGCTTGTCTGTCTGAG	3752459	3752474	7.14E-05	PA3340: hypothetical protein;<br>PA3341: probable transcriptional regulator

TGCTTGTACGCATACG	724610	724625	7.23E-05	PA4280.2: 23SrRNA
TGCTTGTACGCATACG	4791203	4791218	7.23E-05	PA4690.2: 23SrRNA
TGCTTGTACGCATACG	5266731	5266746	7.23E-05	PA5369.2: 23SrRNA
TGCTTGTACGCATACG	6042215	6042230	7.23E-05	PA0668.4: 23SrRNA
TGGTTGTAAGTACAGA	1485968	1485983	7.29E-05	PA1370: hypothetical protein; PA1371: hypothetical protein
TGCTAGTGTGTAAAAC	3546997	3547012	7.41E-05	PA3160: *wzz*, o-antigen chain length regulator
TGCTTGTATCTCAAGG	417508	417523	7.57E-05	PA0372: zinc protease; PA0373: *ftsY*, signal recognition particle receptor
TGCACCTATGTACAAT	5672231	5672246	8.09E-05	PA5036: *gltB*, glutamate synthase large chain precursor
CGGTTTCATGTACAAG	5426167	5426182	8.17E-05	PA4835: *cntM*; PA4834: *cntI*, putative nicotianamine synthase
TGCTTGAATTTAAAAA	1245777	1245792	8.29E-05	PA1151: *imm2*, pyocin S2 immunity protein
TGCTTATAACTATAAG	1692716	1692731	8.35E-05	PA1554: *ccoN1*, cytochrome c oxidase, Cbb3-type
TGTCTGTATGTAAAAA	1400645	1400660	8.40E-05	PA1288: probable outer membrane protein precursor, long chain fatty acid transporter?
TGGTTGTATGTAGATC	3542886	3542901	8.47E-05	PA3158: *wbpB*, UDP-2-acetamido-2-deoxy-d-glucuronic acid 3-dehydrogenase; PA3157: probable acetyltransferase
TCCGTAGATATACAAG	3683450	3683465	8.80E-05	PA3290: *tle1*, hydrolase?
CCCTAGTATATAGAAG	830916	830931	8.87E-05	PA0761: *nadB*, L-aspartate oxidase; PA0762: *algU*, Alginate biosynthesis
TCCTTATATAAAGAAG	3329662	3329677	9.15E-05	
TACTTGAAAACACCAG	6090728	6090743	9.40E-05	PA5412: hypothetical protein
TACGTGGATATGCATG	3450837	3450852	9.44E-05	PA3077: *cprR*, two-component response regulator
TCCTTCTATATGCAAT	2473522	2473537	9.55E-05	PA2247: *bkdA1*, 2-oxoisovalerate dehydrogenase (alpha subunit); PA2246: *bkdR*, transcriptional regulator
TACGGGTATATACCCG	1397225	1397240	9.60E-05	PA1286: major facilitator superfamily transporter
AAGCTTGAACTTACACA	3377524	3377540	5.43E-05	PA3014: *faoA*, fatty-acid oxidation complex alpha-subunit; PA3015: hypothetical protein
ATAGTCGGAAATACAAG	2343116	2343132	7.79E-05	PA2128: *cupA1*, fimbrial subunit
TATTGGTATAGACCAG	597683	597698	3.63E-05	PA0538: *dsbB*, disulfide bond formation protein; PA0539: hypothetical protein
GACATGTATATATACG	5897294	5897309	3.88E-05	PA5238: o-antigen acetylase; PA5237: conserved hypothetical protein
TATTTGCATTTAAAAA	2905126	2905141	3.95E-05	PA2569: hypothetical protein
TCTTTGGATAGACAAA	5747948	5747963	4.81E-05	PA5103: *puuR,* ABC transporter substrate binding protein
CACTTGTCTAGACAAA	3788175	3788190	4.95E-05	PA3381: GntR family transcriptional regulator
TACATGGTTATTCAAA	699967	699982	5.12E-05	PA0643: hypothetical protein; PA0644: hypothetical protein
CATTTCTATATAGAAG	1558933	1558948	5.37E-05	PA1431: *rsaL*, regulatory protein; PA1432: *lasI*
TACTTATAAATAAAAA	5820495	5820510	6.26E-05	PA5170: *arcD*, arginine ornithine antiporter
TATTTATATATTCGAA	2607095	2607110	6.49E-05	PA2359: *sfa3*, probable transcriptional regulator
TATATTCAGATACAAA	213605	213620	6.68E-05	PA0187: hypothetical protein
TGTATGAATGTACAGT	2516349	2516364	6.86E-05	PA2288: hypothetical protein
TGTATGTAAATACAGT	732791	732806	7.09E-05	PA0672: *hemO*; heme oxygenase PA0671: hypothetical protein
TGCATGTGTATGCCAG	5894399	5894414	7.49E-05	PA5236: probable aromatic hydrocarbon reductase
TGCCTTTGTATACAAA	1652031	1652046	7.68E-05	PA1520: GntR family transcriptional regulator; PA1519: probable transporter
TGATTGTATACATAAA	4387175	4387190	8.25E-05	PA3918: *moaC*, molybdopterin biosynthetic protein C
TGTATTTTTATACAGG	514572	514587	8.79E-05	PA0456: cold-shock protein; PA0455: *dbpA*, RNA helicase
TGTATGTAAATGTAAA	4663738	4663753	9.92E-05	PA4167: oxidoreductase; PA4168: *fpvB*, second ferric pyoverdine receptor
TGTTTGTATACAAACA	1746114	1746129	9.94E-05	PA1602: probable oxidoreducatse

TACATGAATGATCAAA	559489	559504	4.24E-05	PA0500: *bioB*, biotin synthase;<br>PA0499: probable pilin assembly chaperone protein
CTGTATATGGATACAGTA	81078	81095	4.63E-06	PA0069: conserved hypothetical protein
CAGGATATGAATTTACCA	5601089	5601106	1.00E-05	PA4986: probable oxidoreductase;<br>PA4985: hypothetical protein
CTGTTAAGGTCTATGCAA	3852568	3852585	1.13E-05	PA3446: NADPH dependent reductase;<br>PA3445: hypothetical protein
CGGGGTATGTATATACCC	1073918	1073935	1.18E-05	PA0993: *cupC2*, pili and flagellar chaperone
CTGGTTATGGGCATACAA	4936915	4936932	1.24E-05	PA4405: hypothetical protein;<br>PA4403: *secA,* secretory protein
CTGTTTCTGGATCTACAG	4360251	4360268	1.48E-05	PA3893: conserved hypothetical protein
CTGCCCATGGATATACAC	4612428	4612445	1.57E-05	PA4124: *hpcB*, homoprotocatechuate 2,3-dioxygenase;<br>PA4123: *hpcC*, 5-carboxy-2-hydroxymuconate semialdehyde dehydrogenase
AAGAATATGTCTATTCAA	883205	883222	2.13E-05	PA0805: hypothetical protein;<br>PA0806: hypothetical protein
CAGTATATTTCGAGACAA	3772701	3772718	2.69E-05	PA3361: *lecB*, fucose-binding lectin PA-IIL, biofilm and cell movement;<br>PA3360: probable secretion protein
CTGTATAAGTAGACAGTA	4052648	4052665	2.69E-05	PA3617: *recA*;<br>PA3619: hypothetical protein
CTCGATCTGTCTCTACAA	5392120	5392137	3.78E-05	PA4806: probable transcriptional regulator;<br>PA4805: probable class III aminotransferase
GTGTATAAATATCCACAA	2228861	2228878	4.50E-05	PA2037: hypothetical protein
ATGTATTTGTATAGATAT	5225915	5225932	5.39E-05	PA4658: hypothetical protein
CTCTTTATCTAAATAAAA	1284353	1284370	5.97E-05	PA1182: probable transcriptional regulator;<br>PA1183: *dctA*, C4-dicarboxylate transport protein
CTGTGAGTGAAGATGCAA	6247728	6247745	6.20E-05	PA5552: *glmU*, glucosamine-1-phosphate acetyltransferase/N-acetylglucosamine-1-phosphate uridyltransferase
CAGTATTTGAAAAAGCAA	2761622	2761639	6.21E-05	PA2461: hypothetical protein
CGGAAGATGGCTAAACAA	5069048	5069065	6.35E-05	PA4526: *pilB*, type 4 fimbrial biogenesis protein PilB
CTGGAAGTGGATATCCAG	4888917	4888934	6.50E-05	PA4360a: hypothetical protein
CTGGATCTGGCAAAACAA	501082	501099	6.74E-05	PA4797: probable transposase
CTGACTCTGGAAATACAG	5003763	5003780	6.74E-05	PA4474: *tldD*, protease
CAGTATCTGCAAGGACAA	5220234	5220251	6.74E-05	PA4653: *cupE6*, adhesin-like protein
CTGGAAATGAATATCCTG	4273643	4273660	6.89E-05	PA3816: *cysE*, O-acetylserine synthase;<br>PA3817: probable methyltransferase;<br>PA3818: *suhB*, extragenic suppressor protein
CTGTACATGAAGGTGCAC	4169578	4169595	7.07E-05	PA3724: *lasB*, elastase;<br>PA3723: probable FMN oxidoreductase
CTGCAGATGAACAGACGA	5562432	5562449	7.07E-05	PA4957: *psd*, phosphatidylserine decarboxylase
CCGTGGCTGAATATGCAA	1058947	1058964	7.07E-05	PA0975: probable radical activating enzyme;<br>PA0976: hypothetical protein
CTGAAGCTGTACGTACAG	1738442	1738459	7.07E-05	PA1596: *htpG*, heat shock protein;<br>PA1597: hypothetical protein
CTGTACATGAAGGGACCA	2654275	2654292	7.07E-05	PA2397: *pvdE*, pyoverdine transporter;<br>PA2398: *fpvA*, ferripyoverdine receptor
CTGGATATGGAAACCCAG	3679970	3679987	7.07E-05	PA3286: beta-acetoacetyl-acyl carrier synthase;<br>PA3287: hypothetical protein
CCGCGCAAGAATATACAA	1980983	1981000	7.44E-05	PA1822: *fimL*, hypothetical protein
CGGTAGATGTACATGCCG	5303847	5303864	7.44E-05	PA4725: *cbrA*;<br>PA4724.1: conserved hypothetical protein
CTGCAGGCGGATATACAG	5960101	5960118	7.44E-05	PA5294: putative multidrug efflux pump;<br>PA5293: transcriptional regulator
CTGGAGGTGCACATACAG	2027429	2027446	7.44E-05	PA1866: ATP dependent DNA helicase
CGGTATCGGTATAGGCCA	5054512	5054529	7.44E-05	PA4515: hydroxylase;<br>PA4514: *piuA*, probable outer membrane receptor for iron transport
CTGGATCTGGATGGTCAA	3759625	3759642	7.65E-05	PA3347: *hsbA*, biofilm anti-anti-sigma factor
CTGAAGATGCGCATCCAA	1721288	1721305	7.88E-05	PA1582: *sdhD*, succinate dehydrogenase D subunit
CTGGTGGGGTTTATACAA	2762064	2762081	7.88E-05	PA2461: hypothetical protein

CTGAACCTGGATATCCAG	509367	509384	7.88E-05	PA0452: probable stomatin-like protein; PA0451a: amino acid ABC transporter substrate binding protein
CTGGAAAGGCACATACAC	795731	795748	7.88E-05	PA0727: replication initiator protein PA0728: probable bacteriophage integrase
CAGCACATGGATCTGCAA	2549213	2549230	7.88E-05	PA2310: taurine metabolism; PA2311: hypothetical protein; PA2309: hypothetical protein
CCGAGTATGCATGCACAA	4783725	4783742	7.88E-05	PA4275: *nusG*, transcription-antitermination protein; PA4274: 50S ribosomal protein L11
CGGAATATGCATATTCCG	2312407	2312424	8.14E-05	PA2101: hypothetical protein; PA2100: probable transcriptional regulator
CTGGAAATGTAATTTCAT	2843707	2843724	8.14E-05	PA2523: *czcR*, two component response regulator; PA2522: *czcC*, outer membrane protein precursor
CTGAGTATGTACCGTCAA	2964727	2964744	8.14E-05	PA2621: *clpS*, biofilm related gene; PA2620: *clpA*, ATP-binding protease component; PA2622: *cspD*, encoding cold-shock protein
CTGCGTCTGGATGTACCA	1276651	1276668	8.36E-05	PA1176: *napF*, ferredoxin protein; PA1177: *napE*, periplasmic nitrate reductase protein; PA1178: *orpH*, PhoP/Q and low Mg^{2+} inducible outer membrane protein H1 precursor
CTGTACATGGGCCTGCAA	1769581	1769598	8.36E-05	PA1626: major facilitator superfamily transporter
CGGCATGCGTATCTACGA	4380756	4380773	8.36E-05	PA3910: *eddA*, extracellular DNA degradation protein; PA3909: *eddB*, extracellular DNA degradation protein
CTGTACCTGTGGCTGCAA	4723280	4723297	8.36E-05	PA4219: *ampO*; PA4218: *ampP*, regulation of beta-lactamase activity
CTGTAGAGGTAGTCCCAA	5352392	5352409	8.56E-05	PA4764: *fur*, ferric uptake regulation protein; PA4765: *omlA*, outer membrane lipoprotein precursor
CTGGATCTGGATGTTCAC	6038962	6038979	8.56E-05	PA5369: *pstS*, phosphate ABC transporter, periplasmic phosphate-binding protein
CTGGCCATGACTACACAA	4252998	4253015	8.73E-05	PA3793: hypothetical protein; PA3795: probable oxidoreductase
CAGTATTCGTACATCCAG	1294152	1294169	8.93E-05	PA1193: hypothetical protein; PA1191: hypothetical protein
CTGCACATGTTCAAGCAA	1779871	1779888	8.93E-05	PA1635: *kdpC*, potassium transporting ATPase, C chain
CTGTATTCGAATATCCGT	2555567	2555584	9.12E-05	PA2317: probable oxidoreductase; PA2318: hypothetical protein
CCGTCCATGTCGATGCAA	4301478	4301495	9.12E-05	PA3839: probable sodium:sulfate symporter; PA3840: conserved hypothetical protein
CTGTCGATGCCTATCCGA	5924360	5924377	9.12E-05	PA5262: *fims*, cell motility; PA5263: *argH*, argininosuccinate lyase
CTGTTCGTGGATATCCAC	3689962	3689979	9.32E-05	PA3295: probable HIT family protein; PA3296: *phoA*, alkaline phosphatase
CTGGATTCGTACATCCAG	1885705	1885722	9.32E-05	PA1742: *pauD2*, glutamine amidotransferase class I; PA1741: hypothetical protein
CTGCTTATGTATTGGCAC	5308573	5308590	9.44E-05	PA4726.11: *crcZ*, ncRNA
CTGTCCATGTATCACCTA	2004325	2004342	9.57E-05	PA1844: *tse1*; PA1843: *metH*, methionine synthase; PA1846: *cti*, cis/trans isomerase
CGGAATATGGAAAGAAAA	3937412	3937429	9.57E-05	PA3519: hypothetical protein; PA3520: putative periplasmic substrate binding protein
CTGCTTCCGCATGTACAA	3207627	3207644	9.84E-05	PA2854: putative L,D-transpeptidase
CAGAATATGGATATGGGA	3803461	3803478	9.84E-05	PA3397: *fprA*, heme catabolic process; PA3398: probable transcriptional regulator

[a]DNA sequences corresponding to the Phut-I and Phut-II consensus (**TGTA**-N2-**TACA**) are underlined; identical nucleotides are shown in bold. Candidate sites examined by EMSA are indicated in red font.
